# Exploring the Genetic Overlap Between Metabolic Traits and Anorexia Nervosa

**DOI:** 10.1016/j.bpsgos.2025.100678

**Published:** 2025-12-18

**Authors:** Danielle M. Adams, Murray J. Cairns

**Affiliations:** a Centre for Complex Disease and Precision Medicine, School of Biomedical Sciences and Pharmacy, University of Newcastle, Callaghan, New South Wales, Australia; b Precision Medicine Research Program, Hunter Medical Research Institute, Newcastle, New South Wales, Australia

**Keywords:** Anorexia nervosa, Colocalization, Linkage disequilibrium score regression, Local genetic correlation, Metabolic traits, Pleiotropy

## Abstract

**Background:**

Anorexia nervosa (AN) is an eating disorder with complex biology that remains largely uncharacterized. Recent genome-wide association studies have identified genetic associations between metabolic traits and AN that may relate to the underlying pathophysiology of the condition. Moreover, observational studies have identified evidence of dysregulated metabolic traits in AN, with emerging evidence suggesting that some of these findings are also observed in weight-restored individuals. While there is evidence for putative shared genetic factors linking metabolic traits and AN, the biology underpinning these genetic relationships has not been thoroughly investigated.

**Methods:**

To further explore shared genetic architecture between metabolic traits and AN with regional specificity, we investigated spatially localized genetic correlation and Bayesian colocalization between 6 metabolic traits (body mass index, high-density lipoprotein, leptin, fasting insulin, insulin resistance, and type 2 diabetes) (*n* = 30,931–659,316) and AN (*n* = 72,517).

**Results:**

Significant local genetic correlation was identified across 60 regions, between genetic liability to AN and one of the 6 metabolic traits, after Benjamini-Hochberg correction. Three of these regions showed strong evidence of colocalization with a shared variant (posterior probability > 0.8), indicating potential functional mechanisms related to the trait associations for high-density lipoprotein and body mass index.

**Conclusions:**

Using evidence of local genetic correlation and colocalization, we found independent regions of the genome that may determine the genome-wide genetic correlation between metabolic traits and AN and identified specific shared genes which may assist with our mechanistic understanding of the inherent biological link between AN and metabolites.

## Plain Language Summary

Anorexia nervosa is a psychiatric disorder influenced by both genetic and environmental components. Emerging evidence suggests that there may be underlying metabolic dysregulation present in the disorder rather than it solely being a consequence of food restriction. Genome-wide association studies are available that measure the association between genetic variants, metabolic traits, and anorexia nervosa, which can be used to investigate the relationship between these traits. Utilizing these data, we identified genetic variants and regions of the genome that are associated with both metabolic dysregulation and increased risk of anorexia nervosa. These variants may function through genes and explain the biological mechanisms through which these traits are associated.

Anorexia nervosa (AN) is a complex psychiatric disorder influenced by both genetic and environmental components (1). Genetic and observational evidence suggest that this disorder may involve underlying metabolic dysregulation (2). For example, patients with AN often experience elevated high-density lipoprotein (HDL) levels (3) and insulin sensitivity (4) in addition to decreased leptin levels (5,6). In weight-restored patients with AN, HDL and leptin may be elevated above levels in the control population, which could indicate that dysregulation of these metabolic traits is not due solely to food restriction (5,7). Genetic correlation, estimated using linkage disequilibrium score regression (LDSC), also suggests associations between levels of different metabolic traits and AN (8). While these associations are not by themselves indicative of causal relationships, genetic methods of causal inference indicate that these may exist. We recently used genetic evidence to show that fasting insulin (FI) may have a causal influence on liability to AN (9). Although there is growing evidence supporting the relationship between FI, other metabolic traits, and AN, little is known about the specific nature of these relationships. Further understanding of the mechanisms through which these traits are correlated is required to understand their role in AN pathogenesis.

Local genetic correlation is a measure of the shared genetic association between 2 traits within a specific region of the genome (10). This may be used to prioritize regions of the genome relevant to understanding the overlapping genetic architecture between 2 traits. Local genetic correlation and colocalization of metabolic traits and AN have not been reported previously and may reveal novel pleiotropic mechanisms of action between these conditions (11, 12, 13). Colocalization may suggest the presence of vertical pleiotropy, whereby an individual genetic variant functions through one trait to influence another, or horizontal pleiotropy, where a variant influences 2 traits through independent mechanisms (14). Additionally, we can identify genes associated with these traits and variants that may be important to understand the observed genetic correlation between AN and metabolic traits, as described herein. Using these methods, we can generate new data that can be utilized to investigate the nature of the genetic relationship between metabolic traits and AN.

## Methods and Materials

### Study Design

**Figure 1:**
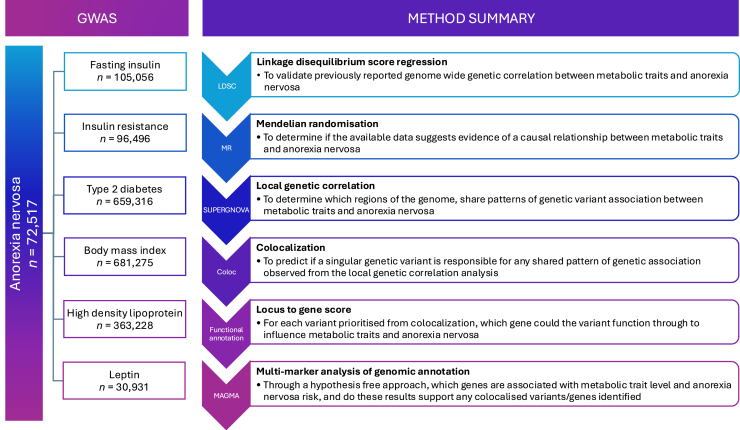
Summary of the methodological pipeline. The sample size and phenotype for each GWAS used is indicated: anorexia nervosa, fasting insulin, insulin resistance, type 2 diabetes, body mass index, high-density lipoprotein, and leptin. Each of the 6 methods (LDSC, MR, SUPERGNOVA, COLOC, functional annotation, and MAGMA) are indicated in order. Beside each method is the unabbreviated name of the method and a summary of the methods’ aim in the context of this study. GWAS*,* genome-wide association study*;* LDSC, linkage disequilibrium score regression; MR, Mendelian randomization.

A summary of the methods used to disentangle the genetic relationship between metabolic traits and AN is presented in this section and illustrated in Figure 1. A glossary of terms is provided in the Supplement to aid in interpretation. In this study, 6 metabolic traits (discussed in Study Datasets) were chosen due to significant genetic correlations with AN (8). Since that study was published, additional genome-wide association studies (GWASs) with increased numbers of measured single nucleotide polymorphism (SNPs) or sample sizes for these traits have become available. Thus, LDSC was performed to confirm that a genome-wide genetic correlation between each metabolic trait and AN was not altered by using alternative GWAS (15,16).

Next, Mendelian randomization was performed to determine whether the available data suggest evidence of a causal relationship between the metabolic traits and AN (17,18). The presence of assumption violations and lack of statistical significance limit the interpretability of these data; however, it is the authors’ belief that null results should still be reported. Thus, details of this analysis can be found in the Supplement and are not further reported in the main text.

Although each metabolic trait is correlated with AN when considering the entire genome, this relationship may not exist for every subset of variants (regions). Local genetic correlation was implemented to quantify the degree of correlation within each specific region for each trait (10). Regions with strong correlations between variant signals may reveal shared biological processes for both traits. Colocalization can be performed to determine the probability that a variant influences both traits rather than separate trait-associated variants in proximity (14). Finally, functional annotation predicts the genes that these variants are likely to function through (19). Targeting the expression of these genes may be useful for modulating both traits in tandem.

To provide additional data to support the association between genes predicted to influence metabolic traits and AN liability, a supplementary analysis was performed using MAGMA (20). This included a gene and gene-set association analysis as well as a meta-analysis with each trait to increase the power of smaller GWASs to detect associations (21). Due to the limitations of this method, it was treated as a supplementary analysis to the colocalization, and thus details about this methodological process and results are primarily reported in the Supplement and are referenced in the main text where applicable.

### Study Datasets

Multiple metabolic disorders have been associated with AN in observational studies (3, 4, 5, 6, 7); however, this association also extends to an inherent genetic relationship. The traits used in this analysis were selected based on significant genetic correlation with AN (*n* = 72,517) previously reported by the AN GWAS publication (8) and were as follows: type 2 diabetes (T2D) (*n* = 659,316) (22), leptin (*n* = 30,931) (23), FI (*n* = 105,056) (24), HDL (*n* = 363,228) (25), body mass index (BMI) (*n* = 681,275) (26), and insulin resistance (homeostatic model assessment for insulin resistance [HOMAIR]) (*n* = 96,496) (27). All GWASs were derived from European ancestry cohorts; thus, LD scores were determined using the 1000 Genomes Project phase 3 European reference panel (28). The HapMap3 reference panel encompasses 1,217,311 SNPs, between 1,009,568 and 1,186,387 of which overlapped with the GWAS described above. Genetic correlations between the 6 metabolic traits and AN were estimated using LDSC as implemented in *ldsc.py* version 1.01 (15,16). Prior to analysis, SNP association data for each trait was munged to enable harmonization between datasets for LDSC using *munge_sumstats.py* restricted to HapMap3 SNPs (29).

### Estimating Local Genetic Correlation Between Metabolic Traits and AN

LDSC estimates the genetic correlation between 2 traits; however, it does not provide specific information about the genetic variants responsible for the association because it utilizes variants throughout the entire genome (30). In contrast, local genetic correlation estimates genetic correlation using variants isolated to a specific region of the genome. These regional correlations were estimated to provide information about which specific loci may give rise to the genome-wide correlation. To estimate the local genetic correlation between the 6 metabolic traits and AN, the SUPERGNOVA (version 1) package was used, which is robust to potential GWAS sample overlap. To estimate the local genetic correlation within each region, the covariance (joint variability) of each trait’s association estimates must be determined. To predict the local genetic covariance, SUPERGNOVA utilizes both the SNP association with each phenotype (GWAS *z* statistic) and the LD between SNPs in a region (eigenvectors of the LD reference panel). These covariance values are then scaled by the SNP heritability (variance of the phenotype explained by each SNP) to determine the regions correlation estimate. This value will be bound by approximately −1 and 1, similar to the genome-wide estimate obtained from LDSC. Local genetic correlations (ρ) surviving a Benjamini-Hochberg false discovery rate (FDR) <.05 were deemed statistically significant. A positive genetic correlation indicates that variant effect size estimates in that locus have proportionally increased association with trait risk, while a negative correlation indicates a proportionally decreased association. This can provide valuable information about the statistical significance of associations across the genome in addition to the directional consistency of these associations. A total of 2,197 mutually independent LD blocks were used as region partitions. These were published by SUPERGNOVA, which used LDetect and LD estimates from the 1000 Genomes Project phase 3 (31).

### Identifying Colocalization Within Loci Exhibiting Local Genetic Correlation

When multiple variants in proximity are associated with different traits, it can be difficult to determine the mechanistic actions of these variants on either trait. These variants may be colocalized, meaning that 2 traits share the same causal variant. However, it is also possible for nearby variants to be associated with 2 different traits, thereby giving the appearance that they might be shared, despite acting through independent mechanisms (32). Identifying local genetic correlation from SUPERGNOVA does not reveal the specific mechanism through which these regions are correlated. To investigate colocalization for the regions of local genetic correlation identified between AN and the 6 metabolic traits of interest, Bayesian colocalization was performed using the COLOC package version 5.2.3, as outlined elsewhere (14). This was performed using GWAS summary statistics for each trait, partitioned by LD-independent blocks, which displayed evidence of association from SUPERGNOVA. COLOC leverages the marginal association of variants with each trait in a region to test 5 competing hypotheses (H_0_–H_4_) encompassing the potential relationships between the variants in each region and the 2 traits of interest. The posterior probabilities (PPs) for two of these hypotheses (H_4_ and H_3_) were of interest in this study.

1. The PP of H_0_ is the probability that variants are not associated with either trait.
2. The PP of H_1_ and H_2_ is the probability that variants are only associated with either trait 1 or 2, respectively.
3. The PP of H_3_ indicates the probability that both traits are associated with separate causal variants within the locus.
4. The PP of H_4_ indicates the probability of colocation between the 2 traits, whereby a region is associated with both traits, and they share an individual causal variant.

Default prior probabilities were used, with p12 used forthwith to denote the prior probability that both traits are colocalized (33). For regions with a high probability of colocalization (H_4_ PP > 80%) (14), the PP value of each hypothesis was plotted in relation to a range of p12 priors. For these regions, which likely harbor colocalization, the PP of each variant being causal is estimated conditioned on H_4_ being true, with these PPs of H_4_ summed to form a 95% credible set. The PP of H_4_ for each individual variant is also reported, where the variant with the highest H_4_ is the variant with the strongest evidence for colocalization. Colocalized variants may represent those that act through vertical or horizontal pleiotropy to influence metabolic traits and AN; however, only vertical pleiotropic relationships represent causal effects of metabolic traits on AN. The standard deviation of the trait phenotype used for COLOC was estimated for each metabolic trait using the minor allele frequency, sample size, and variance in beta estimates. Variants potentially responsible for colocalization were functionally annotated using annotation methods recorded in the Open Targets Platform database (19).

## Results

### Estimating Global and Local Genetic Correlations Between Metabolic Traits and AN

**Figure 2:**
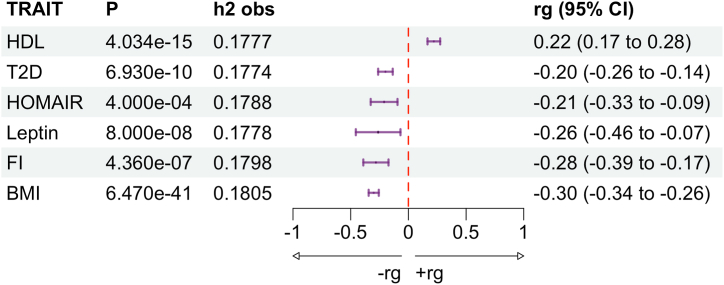
Forest plot of the genome-wide genetic correlation between 6 metabolic traits and anorexia nervosa. Genetic correlation was estimated with linkage disequilibrium score regression. Traits are HDL, T2D, insulin resistance (HOMAIR), leptin, FI, and BMI. Columns indicate the *p* value of the correlation estimate (*p*), the single nucleotide polymorphism heritability of the metabolic trait (*h*^2^), where T2D is on the observed scale, and the genetic correlation (*r*_g_) with 95% CIs of the *r*_g_ estimate are indicated. BMI, body mass index; FI, fasting insulin; HDL, high-density lipoprotein; HOMAIR, homeostatic model assessment for insulin resistance; T2D, type 2 diabetes.

Genetic correlation refers to the correlation between variant association estimates for different phenotypes, which may be positively or negatively associated (34). This may be localized to several loci or be spread throughout the genome. The genome-wide genetic correlations between 6 metabolic traits and AN were estimated using the most recent GWAS using the largest number of variants for each metabolic trait via LDSC. These correlations were reflective of previous results in the literature that used previous GWAS data for each metabolic trait (8) (Figure 2). The genetic correlations (*r*_*g*_s) observed in this study were largely consistent with those reported previously (8), noting that all correlations were within 0.08 units of previous findings. The largest difference occurred for HOMAIR (*r*_g_ = −0.21 vs. −0.29), while the closest results were for BMI (*r*_g_ = −0.30 vs. −0.32), leptin (*r*_g_ = −0.26 vs. −0.24), HDL (*r*_g_ = 0.22 vs. 0.21), and FI (*r*_g_ = −0.28 vs. −0.29). For all traits except HOMAIR, the statistical significance of our AN versus metabolic trait *r*_*g*_ estimates increased compared with the previous analysis with different GWASs.

**Figure 3:**
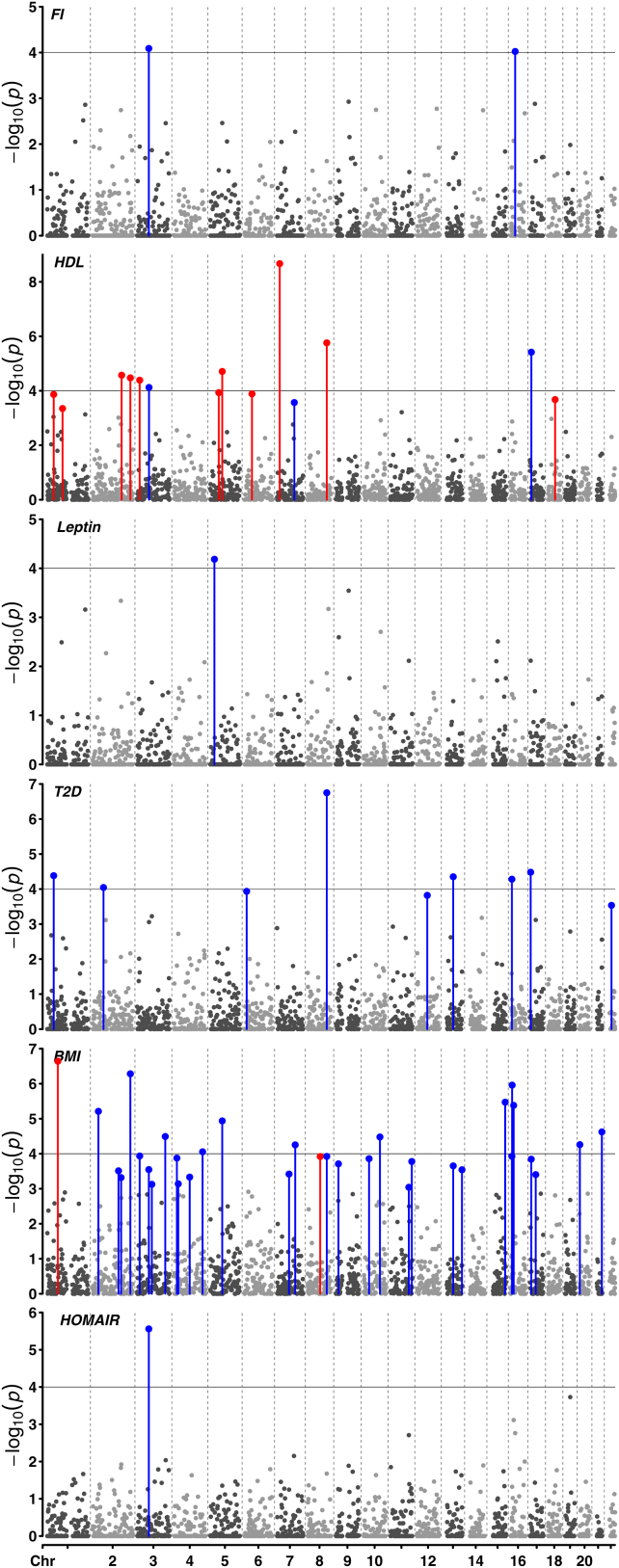
Manhattan plot for local genetic correlations between metabolic traits and anorexia nervosa. Six Manhattan plots are stacked vertically for FI, HDL, leptin, T2D, BMI, and insulin resistance (HOMAIR); all share the same x-axis scale. The x-axis indicates the relative position of each linkage disequilibrium block across all 22 autosomes, where gray dashed vertical lines separate regions/points between each chromosome. The y-axis indicates −log_10_(*p*) for the local genetic correlation estimate within each region. Horizontal line at *p* value 1 × 10^−4^ (y = 4) is indicated. The correlation between each of the 6 traits and anorexia nervosa for each region was estimated with SUPERGNOVA. Red and blue points/lines indicate regions that have a significant positive or negative correlation (Benjamini-Hochberg false discovery rate <5%) between the trait of interest and anorexia nervosa. Dark and light gray points indicate regions with a local genetic correlation that did not pass the significance threshold. Graph generated with CMplot (75) in R. BMI, body mass index; chr, chromosome; FI, fasting insulin; HDL, high-density lipoprotein; HOMAIR, homeostatic model assessment for insulin resistance; T2D, type 2 diabetes.

To further explore the correlation structure between the 6 metabolic traits and AN, we estimated local genetic correlation across segmented regions of the genome (LD blocks approximately 1.6 centimorgans long on average) using SUPERGNOVA (10). This analysis revealed 60 regions of both positive and negative correlation (Benjamini-Hochberg FDR <5%) between metabolic traits and AN (Figure 3 and Table S3), including 9 negatively correlated regions for T2D, 2 for FI, and 1 each for HOMAIR and leptin. Interestingly, AN with BMI and AN with HDL had combinations of both positive and negative local correlations. This included 2 positive regions and 31 negative regions (BMI) and 11 positive regions and 3 negative regions (HDL), respectively. As expected, the direction of the majority of localized genetic correlations reflected the genome-wide direction of genetic correlation between BMI (negative) and HDL (positive) with AN. Eight of the regions that were locally correlated between a metabolic trait and AN were shared with a separate metabolic trait. One region for FI, HOMAIR, and BMI (chromosome 3:70289919–71418327) and HDL, T2D, and BMI (chromosome 8:116095815–117130004) shared an AN correlated region, while the 6 other overlapping regions on chromosomes 1, 2, 3, 5, 13, and 16 were shared between 2 metabolic traits and AN (HDL, BMI, and T2D).

### Identifying Genetic Regions With Evidence for Colocalization Between Metabolic Traits and AN

Colocalization of a locus between 2 traits indicates the presence of 1 shared causal variant (H_4_). Alternatively, there may be shared associations within a locus due to differing causal variants (H_3_). COLOC was used to determine the probability of colocalization between 2 traits within genomic loci prioritized by SUPERGNOVA (14). GWAS association data for each trait were used to update a PP that the 2 traits are colocalized in the region. A PP of H_4_ >80% is considered strong evidence that colocalization may be occurring in the region (14,35); additionally, a PP for H_4_ is calculated for each SNP that indicates the probability that it is the shared causal variant. As the number of variants in the credible set of potentially causal variants increases and the PP of H_4_ decreases, there is less evidence that a specific SNP may be responsible for the colocalization. We specifically analyzed colocalization for each of the 60 regions with significant local genetic correlation from SUPERGNOVA (Table S4). Of these, 3 regions had strong evidence of colocalization (PP H_4_ > 0.8) (Figures S4–S6), while 12 regions had moderate evidence (PP H_4_ > 0.4) (Table 1). Regions of colocalization, assuming a single causal variant, were found for BMI-AN (6 regions), T2D-AN (2 regions), and HDL-AN (4 regions). From these regions, only 2 (HDL and BMI) contained sufficient evidence to prioritize an individual causal SNP, as determined by a low number of SNPs (defined here as <5 SNPs) within the 95% credible set and a high PP (PP H_4_ > 0.8). In addition, 3 loci had strong evidence of a shared association with separate causal variants (PP H_3_ > 0.8), while 5 had moderate evidence (PP H_3_ > 0.4) (Table 2). One of these regions was associated with T2D while the other 4 were associated with BMI.

**Table 1:** Regions With Moderate or Strong Evidence of Colocalization With a Singular Causal Variant

Chr	BP Range	Pheno	H_4_ PP	95% Credible Set, *n*_SNP_s	Prioritized SNP	SNP H_4_ PP	Prioritized Gene
17	7,725,660–8,704,551	HDL	0.975	5	rs8070063	0.716	*TMEM107*
4	16,521,003–18,752,293	BMI	0.866	17	rs11729200	0.248	*LCORL*
21	46,174,981–46,740,019	BMI	0.835	2	rs397092	0.724	*ADARB1*
15	97,092,654–98,496,698	BMI	0.738	19	rs12372926	0.111	*ARRDC4*
2	172,088,943–173,586,201	BMI	0.727	32	rs6738445	0.279	*DYNC1I2*
13	58,248,124–60,085,069	T2D	0.706	85	rs34140906	0.076	*PCDH17*
1	65,010,679–66,773,349	BMI	0.673	17	rs7519259	0.186	*PDE4B*
8	116,095,815–117,130,004	T2D	0.574	61	rs800888	0.107	*TRPS1*
2	175,586,510–177,326,682	HDL	0.557	102	rs34029884	0.168	NA
18	43,881,706–45,313,390	HDL	0.535	68	rs612519	0.048	NA
8	116,095,815–117,130,004	HDL	0.471	35	rs10808475	0.075	*TRPS1*
2	228,457,117–229,368,530	BMI	0.428	34	rs10211055	0.105	*SPHKAP*

Colocalization was performed with COLOC where moderate evidence of colocalization is indicated by a PP of H_4_ > 0.4 and strong evidence by PP > 0.8. The *n*_SNP_s in the credible set was determined by the minimum number of SNPs such that the sum of their H_4_ PPs was >0.95. The prioritized SNP was determined based on the SNP within the region with the largest H_4_ PP. The prioritized gene was determined from the prioritized SNP and Open Targets platform based on the highest L2G score (19,77). Chr indicates the chromosome that the region is located within. BP range indicates the start and end BP positions of the region. Pheno indicates the phenotype colocalized with AN, HDL, BMI, or T2D.

AN, anorexia nervosa; BMI, body mass index; BP, base pair; HDL, high-density lipoprotein; L2G, locus to gene; NA, not available; PP, posterior probability; SNP, single nucleotide polymorphism; T2D, type 2 diabetes.

**Table 2:** Regions With Moderate or Strong Evidence of Genetic Association With Different Causal Variants

Chr	BP Range	Pheno	H_3_ PP
3	70,289,919–71,418,327	BMI	0.987
11	111,985,737–113,103,996	BMI	0.941
22	29,456,360–30,604,102	T2D	0.827
13	58,248,124–60,085,069	BMI	0.574
3	88,725,754–94,202,010	BMI	0.562

Colocalization was performed with COLOC where moderate evidence of different causal variants in the locus is indicated by a PP of H_3_ > 0.4 and strong evidence by PP > 0.8. Chr indicates the chromosome that the region is located within. Pheno indicates the phenotype colocalized with AN, BMI, or T2D. BP range indicates the start and end BP positions of the region.

AN, anorexia nervosa; BMI, body mass index; BP, base pair; PP, posterior probability; T2D, type 2 diabetes.

**Figure 4:**
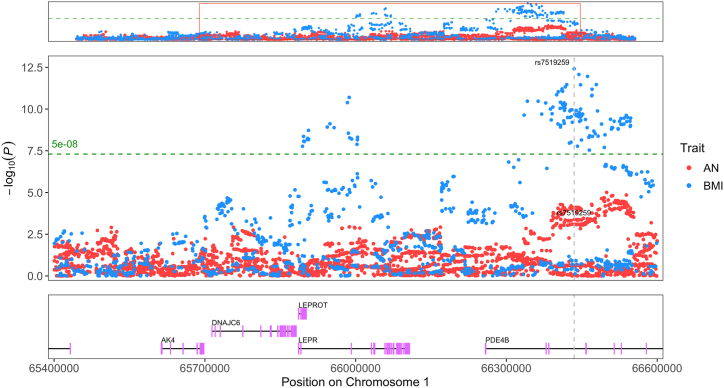
Locus plot displaying a region of colocalization between BMI and AN. Blue and red points indicate SNPs for BMI and AN, respectively. The top of the graph indicates the association pattern of SNPs within the entire linkage disequilibrium–independent locus (chromosome 1:65010679–66773349) of local genetic correlation. This red rectangle overlay in the top panel shows the region displayed in the middle panel. The prioritized SNP (rs7519259) from colocalization (Table 1) is labeled for AN and BMI with text and a gray dashed vertical line. The y-axis indicates −log10(*p*) for the SNP’s association with either trait. The x-axis indicates the base pair location on chromosome 1. The green dashed horizontal line corresponds to the genome-wide significant *p* value (5 × 10^−8^). The bottom graph indicates the location of protein-coding genes within the highlighted region including the prioritized gene from rs7519259, *PDE4B*. Graph generated with topR in R (76). AN, anorexia nervosa; BMI, body mass index; SNP, single nucleotide polymorphism.

**Figure 5:**
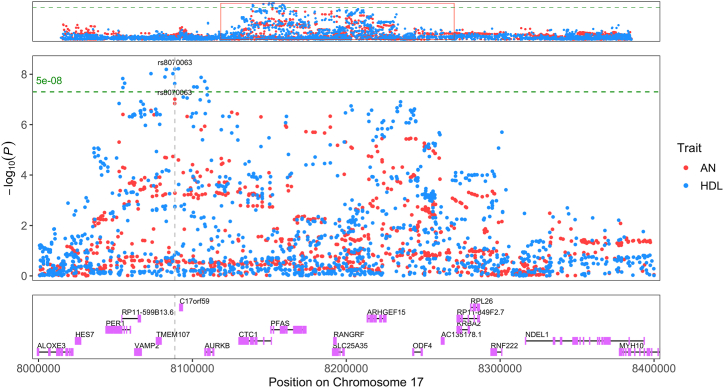
Locus plot displaying a region of colocalization between HDL and AN. Blue and red points indicate SNPs for HDL and AN, respectively. The top of the graph indicates the association pattern of SNPs within the entire linkage disequilibrium–independent locus (chromosome 17:7725660–8704551) of local genetic correlation. This red rectangle overlay in the top panel shows the region displayed in the middle panel. The prioritized SNP (rs8070063) from colocalization (Table 1) is labeled for AN and HDL with text and a gray dashed vertical line. The y-axis indicates −log_10_(*p*) for the SNP’s association with either trait. The x-axis indicates the base pair location on chromosome 17. The green dashed horizontal line corresponds to the genome-wide significant *p* value (5 × 10^−8^). The bottom graph indicates the location of protein-coding genes within the highlighted region including the prioritized gene from rs8070063, *TMEM107*. Graph generated with topR in R (76). AN, anorexia nervosa; HDL, high-density lipoprotein; SNP, single nucleotide polymorphism.

Identifying genes through which genetic variants may function can be used to understand their putative biological mechanisms of action. Variants of interest were identified using Bayes factors obtained via the colocalization approach (highest SNP H_4_ PP) and subsequently used to prioritize a gene based on a high locus to gene (L2G) score for the trait of interest within the Open Targets platform (19). L2G scores are determined from chromatin interaction experiments, quantitative trait loci, distance from transcription start site, in silico functional predictions, and colocalized variants within a GWAS. For the 2 regions (BMI and HDL) that had strong evidence of colocalization and strong evidence to support a specific prioritized SNP, *ADARB1* and *TMEM107* were identified in association with the prioritized causal variants. Furthermore, *PDE4B* (BMI chromosome 1) (Figure 4), *TMEM107* (HDL chromosome 17) (Figure 5), and *ZFHX4* (BMI chromosome 8) were prioritized regions that displayed a different direction of local genetic correlation compared with the genome-wide estimate. The region associated with *PDE4B* (1:65010679–66773349) has moderate evidence of colocalization (H_4_ PP > 0.4); however, the large number of SNPs (17 SNPs) in the 95% credible set and the relatively small PP of the top SNP (PP = 0.19) do not strongly prioritize 1 variant or 1 gene. Thus, other genes located within this region such as *LEPROT* or *LEPR* (Figure 4) may be responsible for any potential colocalization. *LEPROT* and *LEPR* are coexpressed, and while one primary function of LEPROT is to mobilize LEPR to the cell surface, it has other functions including cell surface expression of the growth hormone receptor (36,37), decreasing satiety (38,39), or inhibiting lipolysis (40,41). It should be noted that *PDE4B* was significantly associated with BMI and marginally below the multiple testing correction threshold for an association in the univariate AN gene analysis (*p* = 3.4 × 10^−6^, Bonferroni *p* threshold <2.8 × 10^−6^), which supports evidence that *PDE4B* influences both BMI and AN risk (Tables S9 and S11). Further analysis is required to determine the role of these genes in AN biology.

## Discussion

Here, we estimated local genetic correlations between metabolic traits and AN to provide more granular insights into specific regions of the genome where genetic variants shared patterns of association across traits. Through colocalization, we also prioritized regions where a shared causal variant may be responsible for these associations, and targeting these may be an effective treatment for comorbid AN and metabolic dysregulation. Sixty regions were identified that may have pleiotropic effects between AN and metabolic traits, and of these, there is evidence that a shared causal variant may be responsible for 12 of these associations (H_4_ PP > 0.4).

Here, we identified 3 regions with a local genetic correlation that exhibited evidence for a shared causal variant (H_4_ PP > 0.8) (Table 1 and Table S4). Of these 3 regions, 2 demonstrated sufficient evidence to prioritize a specific lead SNP and a subsequent gene through which it may function. The negatively correlated regions of chromosome 17:7725660–8704551 and 21:46174981–46740019 for HDL and BMI, respectively, prioritized *TMEM107* (Figure 5) and *ADARB1* as genes that may contribute to the shared genetic relationship between metabolic traits and AN. *ADARB1* is responsible for adenosine-to-inosine RNA editing (42), and RNA editing has been associated with BMI (43). This gene has also displayed a potential role in tumor cell inhibition within ovarian cancer cells (44). *TMEM107* encodes a transmembrane protein that functions within the primary cilia transition zone to regulate ciliogenesis and protein composition (45,46) as well as modulate cilia number and length and promote sonic hedgehog signaling (47,48). Currently, there are no approved drugs available that target either of these genes (49); however, in the future, their appropriate regulation may be effective in controlling the co-occurrence of AN and metabolic dysregulation.

Gene mutations within *TMEM107* result in the malfunction of cilia protein and cause ciliopathies (50, 51, 52). Interestingly, most ciliopathies are associated with elevated rates of obesity and metabolic disfunction (53). Cilia are required for the function of melanocortin 4 receptor–expressing neurons in the hypothalamus to regulate metabolic processes (54). Activating this neuron initiates a signal cascade (55) that increases satiety (53,56). Additionally, cilia regulate glucose metabolism in the periphery through regulating adipogenesis (57). Finally, primary cilia can instigate autoimmune responses with immune cells such as CD4+ T and B cells (58). Interestingly, both AN and HDL are associated with altered inflammatory states (59, 60, 61); however, there is insufficient evidence to suggest that TMEM107 functions through inflammatory pathways to influence HDL and AN.

Treating comorbid conditions through targeting a shared overlapping genetic mechanism may be effective to counteract feedback loops between traits and manage co-occurring symptoms. In this study, we used local genetic correlation and conditional analysis to prioritize a variant annotated to *TMEM107* as likely to be causal for both AN risk and HDL levels. MAGMA gene association analysis revealed that *TMEM107* expression is predicted to be associated with risk of AN and levels of HDL (Tables S9, S14, and S27), supporting its role in disease biology. Furthermore, a transcriptome-wide association study (62) suggested that increased *TMEM107* expression would decrease AN risk, and given the direction of association within the local genetic correlation in this region (negative) (Figure 3 and Table S39), it is expected that this would increase HDL levels in conjunction. Patients with AN often experience negative heart conditions due to food restriction–induced malnourishment (63,64). Additionally, HDL has a protective effect against mitigating heart disease (65). In patients with AN, HDL was not found to be associated with illness duration (66), suggesting that targeting *TMEM107* may function to improve both conditions through horizontal pleiotropic mechanisms rather than a feedback loop. Thus, drugs that promote *TMEM107* expression may be more effective for AN treatment, while increasing HDL levels may be beneficial to reduce the damage of malnutrition-induced heart damage.

Although there is insufficient evidence to prioritize *PDE4B* as colocalized between BMI and AN (chromosome 1:65010679–66773349), there is evidence to suggest that it may affect both AN and BMI biology. *PDE4B* is within the phosphodiesterase family, which is involved in axon development and synaptic plasticity via the hydrolysis of cyclic adenosine monophosphate. PDE4 inhibitors have been approved for use in some disorders such as psoriasis due to their anti-inflammatory functions (49,67,68), while this treatment is associated with weight loss as a side effect (69, 70, 71). This may function to reduce BMI; however, decreased appetite may inhibit the treatment of AN. However, one study found that weight-restored mice subjected to an activity-based anorexia study model had reduced *Pde4b* expression relative to control mice (72). This could suggest that inhibition of *PDE4B* expression may have a role in weight restoration and recovery. Additionally, targeting *PDE4B* has been suggested for the combined treatment of binge-eating disorder and alcohol use disorder in a separate mouse model due to its predicted pleiotropic effects on these traits (73).

Although SUPERGNOVA (10) can provide valuable evidence regarding regions displaying local genetic correlation between traits, there are methodological limitations to this approach (10,74). The specific use of genetic boundaries in a hypothesis-free approach for SUPERGNOVA [as well as COLOC (14)] has a large influence on the results of this analysis. Different region boundaries could be used in future analyses to validate variant associations identified here. One particular benefit of the SUPERGNOVA approach applied here is its robustness in the presence of sample overlap, enabling us to utilize larger sample size GWASs containing UK Biobank samples which share samples with the AN GWAS. Although multiple data sources such as expression quantitative trait loci and distance to transcription start site were used to prioritize genes from variants in this analysis, the variant may influence other genes in the locus. A relatively small sample size for some of the GWAS used in this analysis such as leptin and HOMAIR will result in lower estimated SNP heritability within loci and thus the number of significantly associated loci that may be identified. The data analyzed in this study were from European cohorts, with relevant LD-based regional boundaries. Upon the availability of suitably powered datasets from other ancestries, this analysis should be replicated to ensure the applicability of these results to other populations. As with other methods that utilize GWAS association data, the sample size and power of the GWAS used will increase the ability of this approach to detect colocalization because it assumes that variants within this region are significantly associated with either trait. BMI, T2D, and AN all have significantly associated variants; however, the overlap of variants measured between studies will also influence the availability of data. Information from additional sources such as the biological relevance of any prioritized genes should be triangulated with statistical results (such as the PP of SNPs).

### Conclusions

In this study, we identified regions of the genome predicted to be genetically associated between metabolic traits and AN. The association within 2 of these regions was predicted to be due to the influence of a shared causal variant, from which *TMEM107* was identified as likely to influence both AN and HDL. The future availability of drug targets for this gene that increase its expression may increase HDL level while reducing AN risk for the treatment of comorbid AN conditions. Future studies should test the efficacy of such a combined treatment within mouse models to determine their suitability in a clinical setting.

## Supplementary Material

Supplemental Methods, Results, Figures S1–S7, and Tables S1–S8

Tables S9–S40

Key Resources Table
