# Supplementary material for: Exploring the Genetic Overlap Between Metabolic Traits and Anorexia Nervosa
Source: Biol Psychiatry Glob Open Sci. 2025 Dec 18;6(2):100678. doi: 10.1016/j.bpsgos.2025.100678 (PMC12887792; doi:10.1016/j.bpsgos.2025.100678)
Supplement: Supplemental Methods, Results, Figures S1–S7, and Tables S1–S8 [file mmc1.pdf]

SUPPLEMENTARY INFORMATION

Exploring the Genetic Overlap Between Metabolic Traits and Anorexia Nervosa

Adams and Cairns

Supplementary methods and results

Glossary

Anorexia nervosa	Anorexia nervosa is a psychiatric disorder which is influenced by both genetic components and environmental components. Diagnosis is based on the following criteria: persistent weight loss below a healthy weight, inaccurate perception of oneself and anxiety around food.
Bayes factor	The ratio of evidence for two competing hypotheses, to suggest if there is substantially more evidence to support one hypothesis over the other.
Bayesian statistics	Bayesian (in comparison to frequentist) statistical analysis involves updating a prior hypothesis about the probability that an event will occur given the data observed. This results in a posterior probability which represents the probability that the event will occur after considering any available data.
Colocalization	Colocalization is a method used to determine if two events are occurring together. Within this study, it is used to determine if a variant's associations with one trait correlates with its associations to another. A colocalization between gene expression and trait risk may determine that a specific variant is likely to be responsible for changes in both, suggesting that increased gene expression may alter trait risk. However, supplementary analysis would be required to validate the direction and presence of a causal effect.
Causal variant	A causal variant for a particular trait is one which has direct biological influences on trait risk. In contrast, changes to the allele frequency of associated variants may align with trait risk, however they do not influence trait occurrence.
Complex disorder	A complex disorder is an illness which is influenced by both modifiable environmental factors and genetic factors. The degree to which a complex trait is influence by genetics is its heritability (proportion of trait variation explained by genetic factors). Twin studies are typically used to determine the upper bound of the heritability by comparing trait occurrence between monozygotic and dizygotic twins. Complex disorders are also typically polygenic meaning that many different variants influence the trait rather than a few variants that have extremely large effects.
Covariance	Covariance is a measure of the joint variability of two continuous variables. The correlation between two traits is the covariance scaled by the standard deviation of both traits.
Eigenvectors and Eigenvalues	For a given matrix of data values, if there is a vector, which when multiplied by the matrix is the same vector scaled by a number, then the vector is said to be an eigenvector and the number is the eigenvalue. The eigenvector will show the direction of maximum variance in the data. The magnitude of the eigenvectors will indicate how much of the variability in the data is explained by its respective eigenvector.

Functional annotation	Utilising methods to determine the function of genes and proteins. This could involve comparing the sequence of the protein to other genes with known functions, gene knockout studies to determine the consequence of removing or suppressing the gene or interaction studies to determine how the protein binds to the genome.
Genetic correlation	Correlation between two variables is a value bound between -1 and 1 where 0 indicates that changes to one variable are not indicative of any changes to the other variable. A value of 1 indicates that when one value increases the other value will increase by a proportional amount, while -1 indicates that if one value increases the other will decrease by a proportional amount. Values closer to -1 and 1 indicate more consistent changes in the second variable as the first variable increases. Genetic correlation is the correlation between genetic variant associations across two traits.
Genetic overlap	Genetic overlap indicates that a genetic signal (either variant, gene etc) displays the same trends of association (correlation) between two traits. The signal for one trait is said to overlap with the other trait and may occur for a singular signal or signals within a region.
Genome-wide association study	A genome wide association study is a method to quantify the association between variants throughout the genome and a trait of interest. Generally, this method is performed for millions of variants in the genome, while the results for variants not analysed in the study can be predicted from a method called imputation. The more samples available in the study (cases and total sample size) and the higher the heritability of the trait, the more power there is to detect association signals. Due to linkage disequilibrium between variants located close together, some downstream analysis will utilise only one variant per region to avoid confounding from correlated signals. Typically, a genome wide association study will employ a linear regression to estimate the association between a particular genetic variant and the outcome. Additionally, performing multiple tests with the same dataset can increase the change of a false positive so many analyses will use the genome wide significant threshold ($P < 5x10^{-8}$) to indicate variants which are less likely to be a false positive. Genome wide association data will typically contain effect estimates (beta, log odds), standard errors (of the effect estimate), p values (of the effect estimate), effect allele (that the predicted effect is relative to) and variant identifiers (rsIDs).
Harmonisation	Harmonisation is the process in which two datasets derived from GWAS are joined together relative to variant effect alleles. Since there is no consistent method with which each GWAS choses which variant to measure the effect estimates relative to e.g. the minor allele, harmonisation is a vital consideration when comparing the results of two GWAS. Thus, the effect estimate values across the two datasets must be joined relative to the effect alleles for each variant. If each GWAS uses a different effect allele, then one of the values must be multiplied by -1 so that it will now be relative to the respective allele of the other GWAS. During harmonisation variants within linkage disequilibrium may also be removed, as well as strand ambiguous alleles. A strand ambiguous SNP or allele is one with

	insufficient information to determine which strand (forward or reverse) the effect estimate is in relation to.
Horizontal pleiotropy	Horizontal pleiotropy refers to the specific type of pleiotropy where a genetic variant may influence two or more features (such as gene expression, trait risk, protein folding etc), at the same time but through unrelated pathways. For instance, a genetic variant may increase the expression of a microRNA which regulates the expression of protein A and protein B. Where protein A and B have no direct effect on the expression of each other. In this instance, this genetic variant would have horizontal pleiotropic effects on protein A and B since it influences both through a mechanism that is not a cascade of effects.
Linkage disequilibrium	Linkage disequilibrium (LD) is a measure of the increased rate of co-occurrence of SNPs that are located physically close to each other. The closer the SNPs the more likely they are to be inherited together during recombination.
Linkage disequilibrium score regression	Linkage disequilibrium score regression (LDSC) is a bioinformatics method which estimates the genetic correlation between two traits. This utilises GWAS datasets for both traits in addition to linkage scores between variants. LDSC estimates the correlation between the association of each variant with one trait and the other using each available variant in the genome.
Local genetic correlation	Local genetic correlation estimates the genetic correlation between two traits only utilising variants within a limited range (in contrast to LDSC which measures genetic correlation genome wide).
Loci/Locus	A locus is a single genetic variant (locus) or a region of the genome defined by a set of genetic variants (loci). A loci could encapsulate a boundary region around a gene or all variants in LD with a particular variant of interest.
Locus to gene score	The locus to gene score is a value calculated by open target genetics which is used to determine the most likely gene that a variant functions through to influence downstream traits. If a particular variant is strongly linked to a trait and can be functionally mapped to a specific gene, then it is likely that the variant may function through that gene to influence the trait of interest.
Mendelian randomisation	A mendelian randomisation is a type of study designed to emulate a randomised control trial utilising genetic data to provide supporting evidence that a causal relationship may exist between two traits. Genetic variants which are strongly associated with the exposure (treatment) trait are used to proxy the effect of the exposure on the outcome (disease) by analysing the trends of association between the genetic variants with both the exposure and the outcome. To establish evidence of a causal relationship, assumptions about the underlying distribution of the data are made, various methods are then applied to determine if there is evidence that these assumptions have been violated since they cannot be tested directly.
Meta-analysis	A meta-analysis is a process of combining data from similar studies together weighting by differences in sample size. A meta-analysis may also be used within a literature review or similar study to combine the results of multiple studies which have performed

	variations of a similar experiment to determine if the overarching evidence supports a specific conclusion.
Metabolic traits	Metabolic traits are any trait which influences metabolic processes within the body. This includes the regulation of metabolism, energy expenditure and production.
Observational studies	An observational study is a type of study where no intervention is applied, and outcomes are measured across participants. These can be useful to determine trends and correlations between traits, however, may be confounded and thus do not indicate if the relationship between variables is causal.
Pleiotropic mechanisms	This refers to the potential ways (such as horizontal and vertical pleiotropy) that a SNP may influence multiple traits at once.
Posterior inclusion probability	Within colocalization a credible set is generated which represents a set of variants with a particular predefined probability of containing the true causal variant if a causal variant exists in the region. The posterior inclusion probability is the probability that any specific variant belongs within the credible set.
Posterior probability	Within Bayesian statistics a prior probability is attributed to an event based on the expected probability that the event will occur without considering the data within the analysis. This value is then updated to the posterior probability based on the available data.
Single nucleotide polymorphisms	Single nucleotide polymorphisms (SNPs) are base pair changes to the DNA sequence. This could be any change from an A (adenine) to a T (thymine) to a C (cytosine) or a G (guanine). Often genetic variants are SNPs.
SNP heritability	SNP heritability is the variance in the outcome phenotype explained by changes in the SNP (e.g. allele A or allele B). Within a linear model this is represented by the R^2 value.
Vertical pleiotropy	Vertical pleiotropy refers to the specific type of pleiotropy where a genetic variant may influence two or more features (such as gene expression, trait risk protein folding etc), at the same time through a direct pathway from one feature to the next. For instance, the genetic variant may increase gene expression by altering the promoter region of the gene, which results in increased protein expression, which then results in a higher susceptibility for a disease.

Investigating potential causal effects of metabolic traits on anorexia nervosa

Following the genome wide genetic correlation between six metabolic traits and anorexia nervosa (AN) identified from linkage disequilibrium score regression (*Figure 2*), Mendelian randomisation (MR) analysis was performed as a supplementary method. MR was performed using R v3.6.3 (1), TwoSampleMR v0.6.6, and MRPRESSO v1.0 (2). MR uses genetic variants, often single nucleotide polymorphisms (SNPs) as instrumental variables (IVs) to

investigate the causal relationship between an exposure and outcome. It is assumed that the IVs act through vertical pleiotropy on the outcome through the exposure, as opposed to direct effects on the outcome or confounders, and thus any association between the IVs and the outcome is because of the exposure. IVs are chosen based on linkage disequilibrium independence and genome-wide significance, however, some traits such as fasting insulin (FI), insulin resistance (HOMAIR) and leptin contained few IVs thus the less stringent suggestive significance threshold ($P < 1x10^{-5}$) were used for all traits as a point of comparison.

For all models, outlying IVs were detected and removed using the MR Pleiotropy Residual Sum and Outlier (PRESSO) (3, 4) method which measures the residual sum of squares of all IVs to determine outlier status. Following this, all models should contain IVs of sufficient power to detect an association if it exists. This was determined by all sets of IVs having an F statistic greater than 10 (5). The principal method applied to estimate the relationship between each exposure and AN was the inverse variance weighted (IVW) method (3). This method displays the least bias when all IVs used are valid. Adjacent MR methods such as the MR Egger (6), MR weighted median (7), and MR weighted mode (8) may be used as sensitivity analysis to the IVW method to suggest if invalid IVs are biasing these models. Each of these methods has different assumptions about the presence and distribution of IVs, thus consistent results across all methods support the validity of IV assumptions. The MR Egger assumes the no measurement error and instrument strength independent of direct effect assumptions which dictate that IVs cannot have correlated effects on the exposure and confounding variables (9). An I^2 statistic was calculated for each set of IVs which indicated no evidence of no measurement error assumption violation (I^2 near 1) (10). The weighted median is unbiased when most IVs used are valid due to the use of the median value of weighted effect estimates (11, 12). Similarly, the weighted mode is unbiased when no subset of IVs is invalid with a

consistent predicted effect estimate (13). This is known as the plurality valid assumption and stems from the use of probability density functions to determine the effect of the exposure on the outcome (14, 15). Finally, the presence of horizontal pleiotropy can be indirectly tested using two methods: Cochran's Q and the MR Egger intercept. The MR Egger intercept is predicted to account for the indirect effect of IVs on the outcome, thus the deviation of this estimate from 0 suggests the presence of horizontal pleiotropic effects (6). Cochran's Q estimates heterogeneity in the model by analysing the distribution of the residual sum of squares around the predicted values (16, 17). The presence of heterogeneity within the model can be considered a marker of horizontal pleiotropy since the occurrence of only vertical pleiotropy would ensure consistent effect estimates across IVs.

These methods were performed for six metabolic traits (FI, leptin, HOMAIR, body mass index (BMI), type 2 diabetes (T2D) and high-density lipoprotein (HDL)) at two p value thresholds for IV selection ($P < 5x10^{-8}$ and $P < 1x10^{-5}$) which are reported in *Supplementary Table 1 and 2* and shown in *Supplementary Figure 1, 2 and 3*. The BMI IVW model for both p value thresholds were the only models which passed Bonferroni significance ($P < 0.05/6 = 0.0083$). In some instances, such as HDL, the P values of each MR model decrease between the genome wide significance threshold and the suggestively significance threshold for IV selection. However, considering the availability of IVs at the genome-wide significance threshold (nSNPs = 221), any increase in statistical significance using the suggestively significant IVs should be treated cautiously. Similarly, for BMI, the IVW model is more associated at the suggestively significant threshold (OR = 0.65, 95% CI: 0.58-0.73, $P = 1.93x10^{-12}$) compared to the genome-wide significant IVs (OR = 0.65, 95% CI: 0.59-0.73, $P = 4.39x10^{-15}$). Due to the large number of SNPs available in the genome-wide model (nSNPs = 448) there is no justification to use the more lenient p value threshold as this would weaken the MR relevance

assumption. The direction of association remains consistent for all models (OR: IVW = 0.65, weighted median = 0.74, weighted mode = 0.79, MR Egger = 0.91), although, the MR Egger and Weighted mode model have no statistical association ($P > 0.05$). This could indicate a violation of the instrument strength independent of direct effect assumption, multivariate MR might be used to adjust for the association between SNP effects on known confounders of the BMI, AN relationship (18). The Egger intercept suggests some evidence that horizontal pleiotropy is present ($P = 0.03$), and Cochran's Q suggests that heterogeneity is present (Q statistic = 805). Thus, it may be likely that instrumental variable assumption violations are present within this model. Previous studies support a negative correlation between BMI and AN (19, 20) however, there is insufficient evidence to determine if a direct causal relationship is present. MR between HOMAIR, HDL and AN have not been previously published. Previously published MR associations for FI (21), T2D (22) and leptin (23) were not replicated here potentially due to the use of different genome-wide association study datasets. Different datasets were used here to prioritise the number of variants available which subsequently induced some sample overlap between these datasets. Sample overlap can bias MR estimates however will not bias the methods applied within the main text of this study.

Supplementary Table 1: *Mendelian randomisation summary statistics between metabolic traits and anorexia nervosa using genome-wide significant ($P < 5x10^{-8}$) single nucleotide polymorphisms as instrumental variables.*

TRAIT	*FI*	*BMI*	*HDL*	*HOMAIR*	*T2D*
nSNPs	13	448	221	3	110
IVW OR	0.72	0.65	1.08	0.77	0.95
IVW OR lci	0.45	0.58	0.99	0.31	0.91
IVW OR uci	1.16	0.73	1.16	1.87	1.00
IVW P	$1.74x10^{-01}$	$1.93x10^{-12}$	$6.93x10^{-02}$	$5.60x10^{-01}$	$3.86x10^{-02}$
Weighted median OR	0.61	0.74	1.07	1.18	0.93
Weighted median OR lci	0.33	0.63	0.97	0.45	0.87
Weighted median OR uci	1.13	0.87	1.19	3.07	1.00
Weighted median P	$1.18x10^{-01}$	$2.22x10^{-04}$	$1.86x10^{-01}$	$7.35x10^{-01}$	$3.84x10^{-02}$
Weighted mode OR	0.36	0.79	1.10	1.29	0.92
Weighted mode OR lci	0.14	0.57	0.98	0.32	0.82
Weighted mode OR uci	0.92	1.10	1.25	5.10	1.03
Weighted mode P	$5.41x10^{-02}$	$1.59x10^{-01}$	$1.16x10^{-01}$	$7.54x10^{-01}$	$1.38x10^{-01}$
MR Egger OR	1.85	0.91	0.95	60.25	0.98
MR Egger OR lci	0.21	0.66	0.83	0.20	0.87
MR Egger OR uci	16.37	1.27	1.08	18450.29	1.11
MR Egger P	$5.93x10^{-01}$	$5.95x10^{-01}$	$4.19x10^{-01}$	$3.94x10^{-01}$	$7.76x10^{-01}$
Q statistic	15.05	805.71	332.45	2.93	149.14
Q P	$2.38x10^{-01}$	0.00	0.00	$2.31x10^{-01}$	$6.46x10^{-03}$
Egger intercept	-0.02	-0.01	0.01	-0.11	0.00
Egger intercept P	$4.05x10^{-01}$	$3.18x10^{-02}$	$2.04x10^{-02}$	$3.73x10^{-01}$	$5.46x10^{-01}$
I^2	1.00	1.00	1.00	0.99	1.00
F	41.14	74.51	142.98	40.48	70.84

Columns indicate the metabolic trait used as the exposure; FI (fasting insulin), BMI (body mass index), HDL (high-density lipoprotein), HOMAIR (insulin resistance) and T2D (type 2 diabetes). Leptin did not have sufficient genome-wide significant instrumental variables for analysis. Rows indicate the statistics of interest; nSNPs (number of SNPs used in the analysis), OR (odds ratio), lci (lower 95% confidence interval), uci (upper 95% confidence interval) and P (p value). These statistics apply to the four methods used: IVW (inverse variance weighted method), weighted median, weighted mode, and MR Egger. The Q statistic, Egger intercept, I^2 and F statistic are reported. Values have been rounded to two decimal places.

Supplementary Table 2: *Mendelian randomisation summary statistics between metabolic traits and anorexia nervosa using suggestively significant ($P < 1x10^{-5}$) single nucleotide polymorphisms as instrumental variables.*

TRAIT	*FI*	*BMI*	*HDL*	*HOMAIR*	*T2D*	*Leptin*
nSNPs	57	637	380	13	226	25
IVW OR	0.96	0.65	1.10	0.82	0.95	0.99
IVW OR lci	0.79	0.59	1.02	0.57	0.92	0.88
IVW OR uci	1.17	0.73	1.18	1.18	0.99	1.11
IVW P	$6.92x10^{-01}$	$4.39x10^{-15}$	$1.51x10^{-02}$	$2.91x10^{-01}$	$2.45x10^{-02}$	$8.91x10^{-01}$
Weighted median OR	1.05	0.75	1.08	0.73	0.95	0.96
Weighted median OR lci	0.81	0.65	0.98	0.46	0.90	0.82
Weighted median OR uci	1.36	0.87	1.19	1.15	1.01	1.12
Weighted median P	$7.07x10^{-01}$	$1.94x10^{-04}$	$1.19x10^{-01}$	$1.77x10^{-01}$	$1.01x10^{-01}$	$5.74x10^{-01}$
Weighted mode OR	1.08	0.83	1.11	0.71	0.93	1.00
Weighted mode OR lci	0.73	0.58	1.00	0.45	0.84	0.76
Weighted mode OR uci	1.62	1.17	1.23	1.12	1.02	1.33
Weighted mode P	$6.97x10^{-01}$	$2.88x10^{-01}$	$5.30x10^{-02}$	$1.62x10^{-01}$	$1.34x10^{-01}$	$9.74x10^{-01}$
MR Egger OR	1.19	0.86	0.95	0.70	0.96	0.86
MR Egger OR lci	0.86	0.65	0.85	0.40	0.87	0.58
MR Egger OR uci	1.67	1.14	1.08	1.24	1.06	1.26
MR Egger P	$2.99x10^{-01}$	$2.95x10^{-01}$	$4.55x10^{-01}$	$2.46x10^{-01}$	$3.98x10^{-01}$	$4.42x10^{-01}$
Q statistic	87.83	1056.38	590.43	15.62	301.28	28.83
Q P	$4.21x10^{-03}$	$2.07x10^{-23}$	0.00	$2.09x10^{-01}$	$5.09x10^{-04}$	$2.27x10^{-01}$
Egger intercept	-0.01	0.00	0.00	0.01	0.00	0.01
Egger intercept P	$1.18x10^{-01}$	$3.89x10^{-02}$	$4.57x10^{-03}$	$4.81x10^{-01}$	$9.29x10^{-01}$	$4.45x10^{-01}$
I^2	1.00	1.00	1.00	1.00	1.00	1.00
F	26.61	60.38	92.55	25.85	46.39	23.27

Columns indicate the metabolic trait used as the exposure; FI (fasting insulin), BMI (body mass index), HDL (high-density lipoprotein), HOMAIR (insulin resistance), T2D (type 2 diabetes) and leptin. Rows indicate the statistics of interest; nSNPs (number of SNPs used in the analysis), OR (odds ratio), lci (lower 95% confidence interval), uci (upper 95% confidence interval) and P (p value). These statistics apply to the four methods used: IVW (inverse variance weighted method), weighted median, weighted mode, and MR Egger. The Q statistic, Egger intercept, I^2 and F statistic are reported. Values have been rounded to two decimal places.

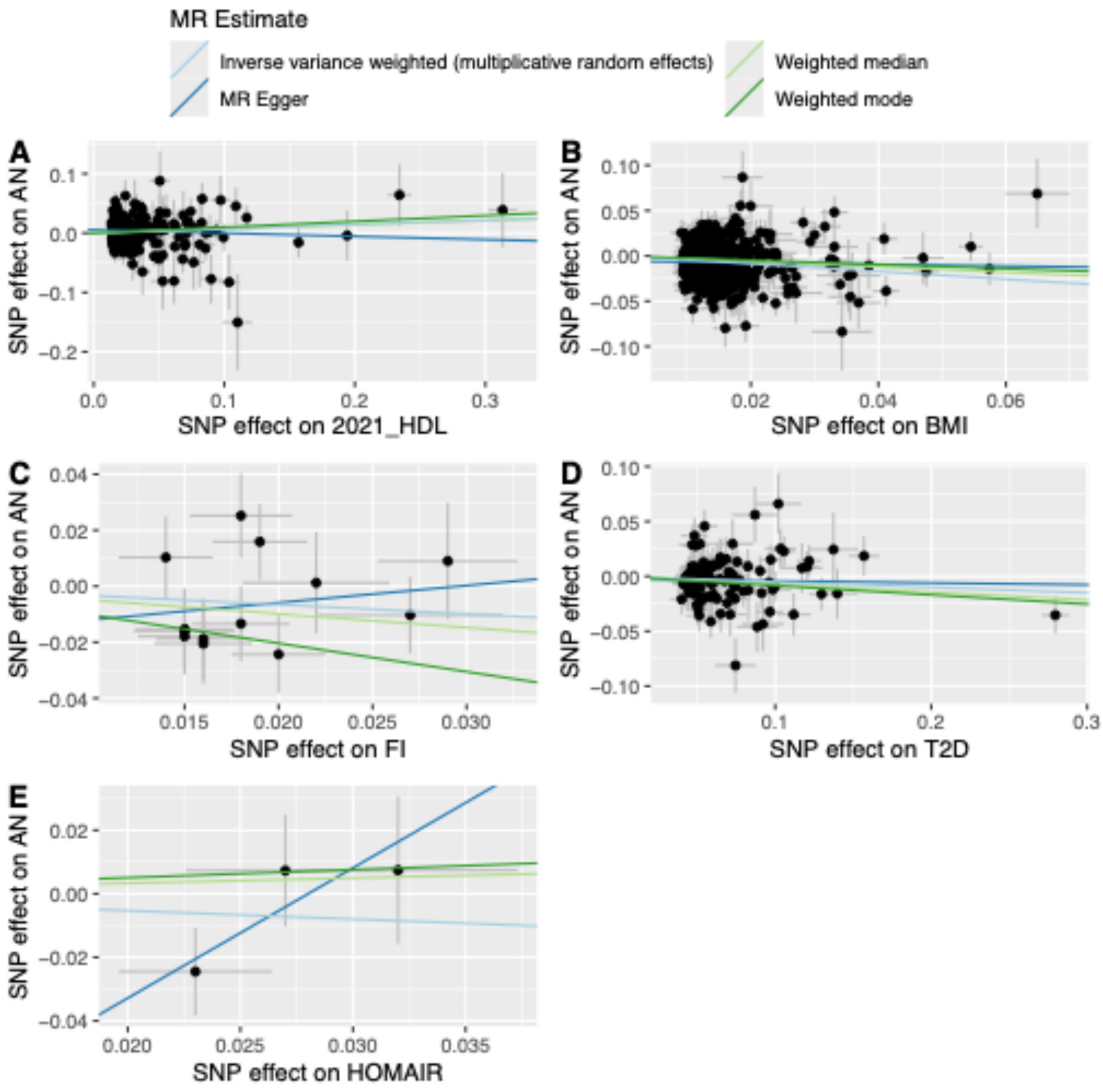

Supplementary Figure 1: *Mendelian randomisation scatter plots using genome-wide significant ($P < 5x10^{-8}$) single nucleotide polymorphisms as instrumental variables. Each graph is for a different exposure* ***A:*** *HDL (high-density lipoprotein),* ***B:*** *BMI (body mass index),* ***C:*** *FI (fasting insulin),* ***D:*** *T2D (type 2 diabetes), and* ***E:*** *HOMAIR (insulin resistance). Leptin did not have sufficient genome-wide significant instrumental variables for analysis. The legend indicates the estimated association for each of the four methods used: Inverse variance weighted (multivariate random effects)- light blue, weighted median- light green, weighted mode- dark green, and MR Egger- dark blue. Each point represents an instrumental variable, the x-axis reflects the SNPs effect on the metabolic trait and the y-axis shows the SNPs effect on anorexia nervosa (AN). Vertical and horizontal lines from each point show the standard errors of the effect estimates. Full summary data is shown in Supplementary Table 1.*

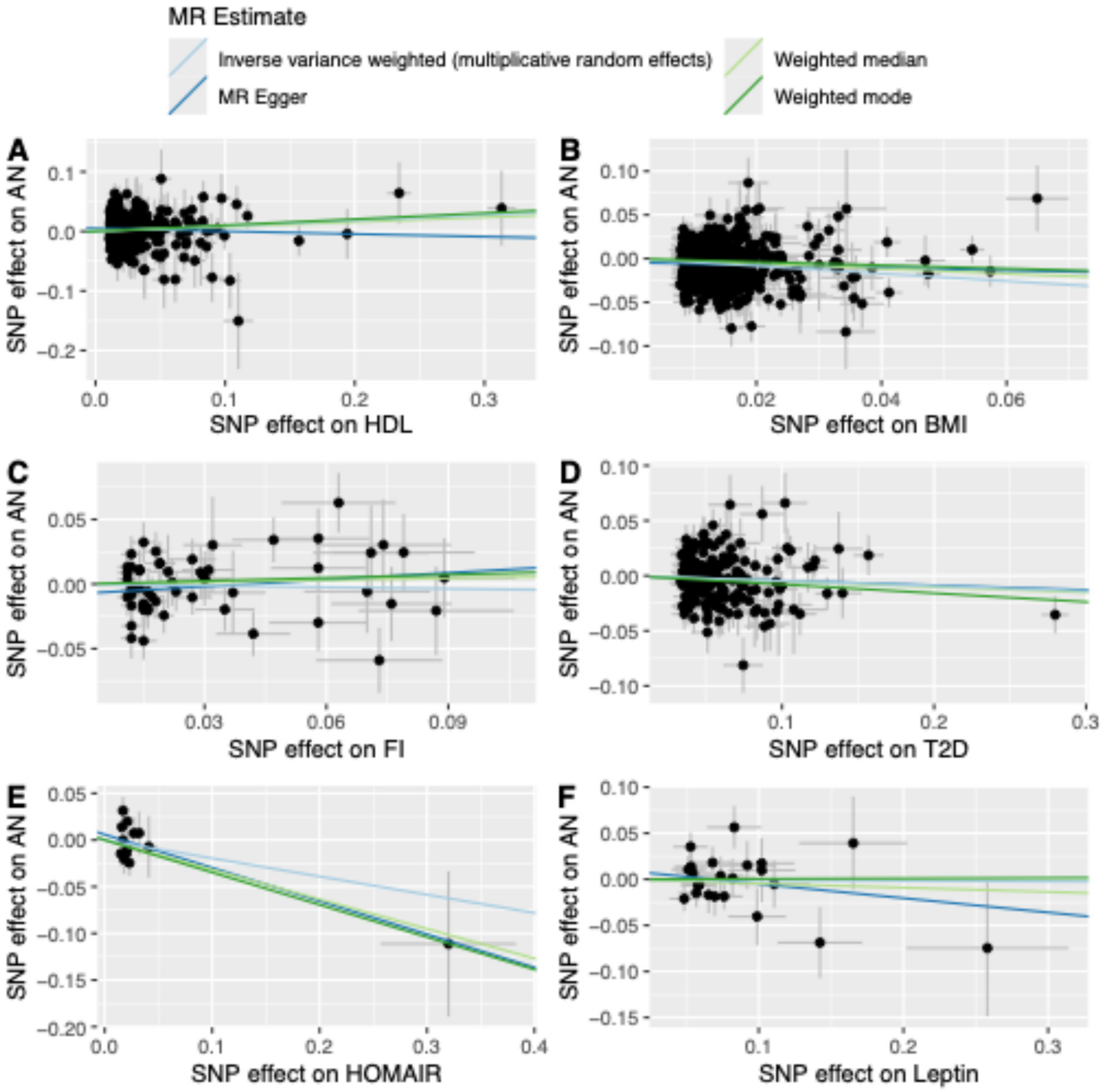

Supplementary Figure 2: *Mendelian randomisation scatter plots using suggestively significant (P < 1x10^{-5}) single nucleotide polymorphisms as instrumental variables. Each graph is for a different exposure* ***A:*** *HDL (high-density lipoprotein),* ***B:*** *BMI (body mass index),* ***C:*** *FI (fasting insulin),* ***D:*** *T2D (type 2 diabetes),* ***E:*** *HOMAIR (insulin resistance) and* ***F:*** *Leptin. The legend indicates the estimated association for each of the four methods used: Inverse variance weighted (multivariate random effects)- light blue, weighted median- light green, weighted mode- dark green, and MR Egger- dark blue. Each point represents an instrumental variable, the x-axis reflects the SNPs effect on the metabolic trait and the y-axis shows the SNPs effect on anorexia nervosa (AN). Vertical and horizontal lines from each point show the standard errors of the effect estimates. Full summary data is shown in Supplementary Table 2.*

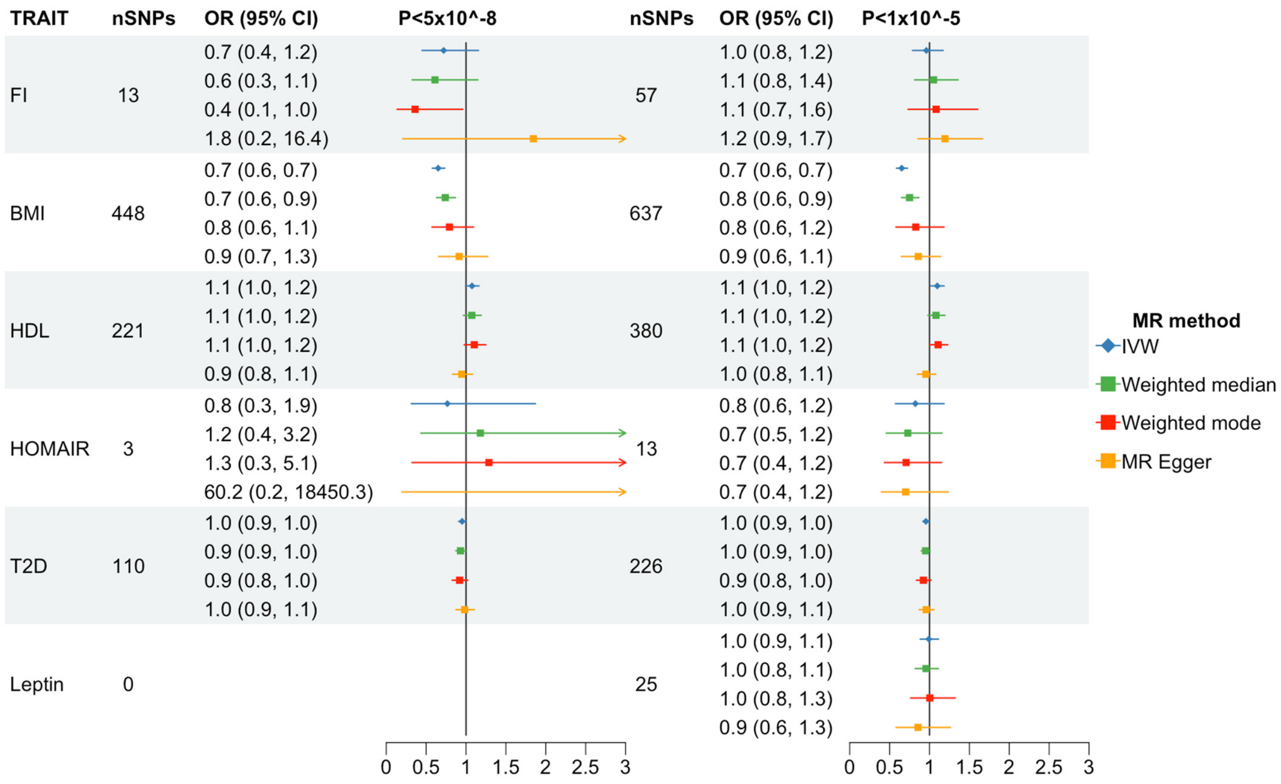

Supplementary Figure 3: *Forest plot summarising the estimated association between metabolic traits and anorexia nervosa using Mendelian randomisation at two p value thresholds. Data for six metabolic traits (TRAIT) are grouped into rows; FI (fasting insulin), BMI (body mass index), HDL (high-density lipoprotein), HOMAIR (insulin resistance), T2D (type 2 diabetes) and leptin. nSNPs indicates the number of single nucleotide polymorphisms (SNPs) available as instrumental variables for either the genome-wide significant p value threshold ($P < 5x10^{-8}$) or the suggestively significant threshold ($P < 1x10^{-5}$). nSNPs and OR (95% CI)- odds ratio with 95% confidence interval is indicated to the left of the graphed estimates. The legend indicates the four methods used; Inverse variance weighted (IVW)- blue, weighted median- green, weighted mode- red, and MR Egger- orange. The x-axis indicates the odds ratio of the estimate, and the y-axis groups the estimates into MR method and TRAIT. The x-axis is constrained from 0 to 3. 1 indicates no association between traits. An arrow on a line or no point estimate indicates that the end of the 95% confidence interval or point estimate exceeds the bounds of 3. Supplementary Table 1 and 2 contain full summary data relevant to this forest plot.*

Supplementary Table 3: *Summary of Benjamini-Hochberg significantly correlated genomic regions determined by SUPERGNOVA.*

pheno	*chr*	*start*	*end*	*ρ*	*corr*	*var*	*p*	*m*
T2D	6	13389521	14618107	-1.56×10^{-04}	-1.79	1.63×10^{-09}	1.16×10^{-04}	1688
BMI	20	1899473	3081680	-2.32×10^{-04}	-1.50	3.32×10^{-09}	5.50×10^{-05}	946
T2D	1	39537291	40933221	-1.73×10^{-04}	-1.45	1.78×10^{-09}	4.15×10^{-05}	1282
FI	16	27446054	29023966	-2.59×10^{-04}	-1.39	4.41×10^{-09}	$9.48\times10\text{-}^{05}$	1113
BMI	4	172737462	173760563	-1.52×10^{-04}	-1.29	1.50×10^{-09}	8.81×10^{-05}	636
Leptin	5	26185302	26849079	-5.36×10^{-04}	-1.23	1.80×10^{-08}	6.53×10^{-05}	1305
BMI	3	170159134	171311936	-2.09×10^{-04}	-1.22	2.53×10^{-09}	3.23×10^{-05}	691
BMI	4	94230511	95596188	-1.76×10^{-04}	-1.22	2.53×10^{-09}	4.66×10^{-04}	833
BMI	13	111434814	113266084	-2.75×10^{-04}	-1.21	5.75×10^{-09}	2.87×10^{-04}	1311
BMI	3	14816745	16661587	-2.58×10^{-04}	-1.18	4.49×10^{-09}	1.16×10^{-04}	1272
HDL	3	71421152	73238935	-3.41×10^{-04}	-1.16	7.43e-09	7.55e-05	3130
BMI	9	14714681	16054406	-2.58×10^{-04}	-1.16	4.78×10^{-09}	1.95×10^{-04}	1081
T2D	8	116095815	117130004	-1.33×10^{-04}	-1.14	6.54×10^{-10}	1.78×10^{-07}	867
BMI	15	97092654	98496698	-3.54×10^{-04}	-1.12	5.79×10^{-09}	3.37×10^{-06}	904
BMI	16	7525825	8236887	-2.51×10^{-04}	-1.12	4.26×10^{-09}	1.19×10^{-04}	829
HDL	7	106052873	107305328	-2.11×10^{-04}	-1.10	3.37e-09	2.69e-04	1931
T2D	16	7525825	8236887	-1.98×10^{-04}	-1.10	2.41×10^{-09}	5.24×10^{-05}	1922
T2D	13	58248124	60085069	-2.53×10^{-04}	-1.08	3.84×10^{-09}	4.47×10^{-05}	3142
BMI	21	46174981	46740019	-2.97×10^{-04}	-1.06	4.96×10^{-09}	2.38×10^{-05}	392
FI	3	70289919	71418327	-3.64×10^{-04}	-1.05	8.55×10^{-09}	8.15×10^{-05}	1214
T2D	17	3426133	4321755	-1.24×10^{-04}	-1.04	8.97×10^{-10}	3.31×10^{-05}	860
BMI	10	99554895	100240681	$-2.25\times10\text{-}^{04}$	-1.04	2.95×10^{-09}	3.33×10^{-05}	470
BMI	2	34280335	35607183	-2.65×10^{-04}	-1.03	3.44×10^{-09}	6.15×10^{-06}	1037
T2D	2	65228236	66374803	-1.76×10^{-04}	-1.03	2.03×10^{-09}	9.04×10^{-05}	1808
BMI	11	129839528	130892029	-2.36×10^{-04}	-1.00	3.93×10^{-09}	1.67×10^{-04}	618
BMI	3	88725754	94202010	-3.30×10^{-04}	-0.97	9.57×10^{-09}	7.48×10^{-04}	819
HOMAIR	3	70289919	71418327	-5.54×10^{-04}	-0.96	1.40×10^{-08}	2.75×10^{-06}	665
BMI	2	172088943	173586201	-2.24×10^{-04}	-0.95	4.12×10^{-09}	4.80×10^{-04}	903
BMI	13	58248124	60085069	-4.84×10^{-04}	-0.94	1.72×10^{-08}	2.23×10^{-04}	1556
T2D	22	29456360	30604102	-1.99×10^{-04}	-0.93	3.01×10^{-09}	2.93×10^{-04}	1387
BMI	16	18065944	20054371	-4.43×10^{-04}	-0.92	9.26×10^{-09}	4.17×10^{-06}	682
T2D	12	61740561	63158569	-1.72×10^{-04}	-0.92	2.07×10^{-09}	1.51×10^{-04}	2398
BMI	16	8962907	9658861	-2.95×10^{-04}	-0.92	3.67×10^{-09}	1.10×10^{-06}	540
BMI	7	111450919	113336970	-2.61×10^{-04}	-0.91	4.18×10^{-09}	5.59×10^{-05}	1005

BMI	4	24993167	26468997	-2.65x10^{-04}	-0.91	6.14x10^{-09}	7.25x10^{-04}	962
BMI	17	6976284	7723513	-1.80x10^{-04}	-0.91	2.23x10^{-09}	1.43x10^{-04}	263
BMI	7	73997700	76550299	-2.57x10^{-04}	-0.90	5.22x10^{-09}	3.81x10^{-04}	403
BMI	10	33656119	36017592	-3.09x10^{-04}	-0.89	6.59x10^{-09}	1.38x10^{-04}	1435
HDL	17	7725660	8704551	-3.77x10^{-04}	-0.84	6.68e-09	3.82e-06	1410
BMI	2	156568806	158165818	-2.13x10^{-04}	-0.82	3.47x10^{-09}	3.08x10^{-04}	723
BMI	2	228457117	229368530	-2.73x10^{-04}	-0.82	2.95x10^{-09}	5.22x10^{-07}	694
BMI	4	16521003	18752293	-3.76x10^{-04}	-0.81	9.68x10^{-09}	1.33x10^{-04}	1496
BMI	5	73508509	75240469	-4.02x10^{-04}	-0.77	8.39x10^{-09}	1.16x10^{-05}	1158
BMI	17	34393296	35725997	-2.49x10^{-04}	-0.76	4.93x10^{-09}	3.95x10^{-04}	418
BMI	11	111985737	113103996	-2.16x10^{-04}	-0.74	4.25x10^{-09}	9.09x10^{-04}	884
BMI	8	116095815	117130004	-1.91x10^{-04}	-0.67	2.45x10^{-09}	1.19x10^{-04}	558
BMI	3	70289919	71418327	-3.11x10^{-04}	-0.59	7.36x10^{-09}	2.84x10^{-04}	627
HDL	5	52662483	53513531	2.05x10^{-04}	0.85	2.84e-09	1.16e-04	1779
HDL	1	93352491	94451007	1.87x10^{-04}	0.86	2.844-09	4.48e-04	1612
BMI	1	65010679	66773349	4.02x10^{-04}	0.88	6.03x10^{-09}	2.28x10^{-07}	1186
HDL	8	116095815	117130004	4.56x10^{-04}	0.88	9.11e-09	1.73e-06	1014
HDL	3	14816745	16661587	3.21x10^{-04}	0.89	6.12e-09	4.08e-05	3395
HDL	6	45788111	46556325	1.69x10^{-04}	0.89	1.95e-09	1.29e-04	1154
HDL	2	228457117	229368530	2.61x10^{-04}	0.90	3.96e-09	3.36e-05	2307
HDL	7	17279294	18861958	5.17x10^{-04}	0.92	7.46e-09	2.14e-09	2504
HDL	5	73508509	75240469	2.88x10^{-04}	0.92	4.55e-09	1.95e-05	3042
HDL	18	43881706	45313390	2.94x10^{-04}	1.08	6.33e-09	2.10e-04	2442
HDL	2	175586510	177326682	2.83x10^{-04}	1.15	4.55e-09	2.68e-05	2582
HDL	1	39537291	40933221	4.58x10^{-04}	1.25	1.44e-08	1.36e-04	1818
BMI	8	76295292	78015335	2.64x10^{-04}	1.30	4.71x10^{-09}	1.20x10^{-04}	1057

Each row represents a genomic region with genetic variants in linkage disequilibrium. Columns indicate; pheno (phenotype which is correlated with anorexia nervosa within this region), chr (chromosome), start (first base pair location within the chromosome for this region), end (last base pair location within the chromosome for this region), ρ (estimated local genetic covariance), corr (estimated local genetic correlation), var (variance of the estimated genetic covariance), p (p value of local genetic covariance), m (number of SNPs in this region). Six metabolic traits are; FI (fasting insulin), BMI (body mass index), HDL (high-density lipoprotein), HOMAIR (insulin resistance), T2D (type 2 diabetes) and leptin. Full results are provided in Supplementary Table 35 – Supplementary Table 40.

Supplementary Table 4: *Colocalization results generated from COLOC for regions with evidence of local genetic correlation.*

chr	*start*	*end*	*pheno*	H_4 *PP*	H_3 *PP*	H_2 *PP*	H_1 *PP*	H_0 *PP*
17	**7725660**	**8704551**	**HDL**	**0.974**	**0.025**	**0.000**	**0.001**	**0.000**
4	**16521003**	**18752293**	**BMI**	**0.866**	**0.109**	**0.000**	**0.025**	**0.000**
21	**46174981**	**46740019**	**BMI**	**0.835**	**0.043**	**0.000**	**0.121**	**0.000**
15	97092654	98496698	BMI	0.738	0.118	0.000	0.144	0.000
2	172088943	173586201	BMI	0.727	0.081	0.000	0.192	0.000
13	58248124	60085069	T2D	0.706	0.203	0.071	0.015	0.005
1	65010679	66773349	BMI	0.673	0.205	0.000	0.122	0.000
8	116095815	117130004	T2D	0.574	0.082	0.012	0.291	0.041
2	175586510	177326682	HDL	0.557	0.161	0.259	0.009	0.014
18	43881706	45313390	HDL	0.535	0.142	0.026	0.252	0.045
8	116095815	117130004	HDL	0.471	0.143	0.000	0.386	0.000
2	228457117	229368530	BMI	0.428	0.048	0.000	0.524	0.000
3	88725754	94202010	BMI	0.355	0.562	0.000	0.083	0.000
16	8962907	9658861	BMI	0.342	0.207	0.000	0.451	0.000
13	58248124	60085069	BMI	0.338	0.574	0.000	0.088	0.000
11	129839528	130892029	BMI	0.329	0.033	0.000	0.638	0.000
2	228457117	229368530	HDL	0.294	0.390	0.049	0.237	0.030
20	1899473	3081680	BMI	0.255	0.164	0.000	0.579	0.001
2	156568806	158165818	BMI	0.233	0.044	0.000	0.722	0.001
16	18065944	20054371	BMI	0.225	0.154	0.000	0.621	0.000
17	3426133	4321755	T2D	0.208	0.039	0.000	0.753	0.000
4	24993167	26468997	BMI	0.169	0.066	0.000	0.765	0.000
17	34393296	35725997	BMI	0.146	0.062	0.000	0.792	0.000
7	**111450919**	**113336970**	**BMI**	**0.143**	**0.058**	**0.000**	**0.800**	**0.000**
2	34280335	35607183	BMI	0.132	0.091	0.000	0.778	0.000
22	**29456360**	**30604102**	**T2D**	**0.128**	**0.827**	**0.001**	**0.043**	**0.000**
3	**14816745**	**16661587**	**BMI**	**0.121**	**0.046**	**0.000**	**0.832**	**0.000**
7	106052873	107305328	HDL	0.100	0.105	0.000	0.793	0.001
10	99554895	100240681	BMI	0.095	0.170	0.000	0.735	0.000
5	26185302	26849079	Leptin	0.092	0.084	0.018	0.661	0.145
1	**39537291**	**40933221**	**T2D**	**0.058**	**0.050**	**0.000**	**0.892**	**0.000**
17	6976284	7723513	BMI	0.056	0.035	0.008	0.733	0.168
11	**111985737**	**113103996**	**BMI**	**0.047**	**0.941**	**0.001**	**0.010**	**0.000**
12	**61740561**	**63158569**	**T2D**	**0.047**	**0.107**	**0.823**	**0.003**	**0.021**
2	**65228236**	**66374803**	**T2D**	**0.046**	**0.109**	**0.000**	**0.845**	**0.000**

3	71421152	73238935	HDL	0.037	0.251	0.001	0.709	0.002
3	14816745	16661587	HDL	0.036	0.186	0.000	0.777	0.000
3	**170159134**	**171311936**	**BMI**	**0.032**	**0.026**	**0.000**	**0.942**	**0.000**
5	**52662483**	**53513531**	**HDL**	**0.026**	**0.123**	**0.000**	**0.850**	**0.000**
8	**76295292**	**78015335**	**BMI**	**0.025**	**0.042**	**0.000**	**0.933**	**0.000**
16	7525825	8236887	T2D	0.021	0.044	0.158	0.170	0.606
1	**39537291**	**40933221**	**HDL**	**0.018**	**0.089**	**0.000**	**0.893**	**0.000**
4	**172737462**	**173760563**	**BMI**	**0.017**	**0.037**	**0.000**	**0.941**	**0.004**
16	7525825	8236887	BMI	0.017	0.020	0.106	0.138	0.718
9	**14714681**	**16054406**	**BMI**	**0.016**	**0.061**	**0.000**	**0.924**	**0.000**
4	**94230511**	**95596188**	**BMI**	**0.013**	**0.055**	**0.000**	**0.932**	**0.000**
3	**70289919**	**71418327**	**HOMAIR**	**0.011**	**0.030**	**0.952**	**0.000**	**0.007**
5	**73508509**	**75240469**	**BMI**	**0.010**	**0.064**	**0.000**	**0.926**	**0.000**
7	**73997700**	**76550299**	**BMI**	**0.010**	**0.101**	**0.000**	**0.889**	**0.000**
5	73508509	75240469	HDL	0.009	0.198	0.000	0.793	0.000
10	33656119	36017592	BMI	0.008	0.375	0.000	0.617	0.000
3	**70289919**	**71418327**	**FI**	**0.007**	**0.051**	**0.936**	**0.000**	**0.005**
13	**111434814**	**113266084**	**BMI**	**0.007**	**0.054**	**0.000**	**0.939**	**0.000**
7	**17279294**	**18861958**	**HDL**	**0.006**	**0.148**	**0.000**	**0.845**	**0.000**
8	**116095815**	**117130004**	**BMI**	**0.006**	**0.156**	**0.000**	**0.837**	**0.000**
6	45788111	46556325	HDL	0.004	0.060	0.394	0.071	0.471
6	13389521	14618107	T2D	0.004	0.009	0.155	0.048	0.783
16	**27446054**	**29023966**	**FI**	**0.002**	**0.003**	**0.048**	**0.057**	**0.890**
3	**70289919**	**71418327**	**BMI**	**0.001**	**0.987**	**0.000**	**0.012**	**0.000**

Regions for which colocalization was performed are indicated in Supplementary Table 3. Each row shows the colocalization results for one region with local genetic correlation, in descending order based on H_4 PP. Columns indicate pheno (phenotype which is correlated with anorexia nervosa within this region), chr (chromosome), start (first base pair location within the chromosome for this region), end (last base pair location within the chromosome for this region), H_4 PP (posterior probability for the H_4 hypothesis that colocalization is present), H_3 PP (posterior probability for the hypothesis that both traits are associated with different causal variants), H_2 PP (posterior probability that variants are only associated with trait 2), H_1 PP (posterior probability that variants are only associated with trait 1 and H_0 PP (posterior probability that variants are not associated with either trait. Bold text indicates regions with strong (PP > 0.8) evidence of one of the hypotheses. Phenotypes are FI (fasting insulin), BMI (body mass index), HDL (high-density lipoprotein), HOMAIR (insulin resistance), T2D (type 2 diabetes) and leptin.

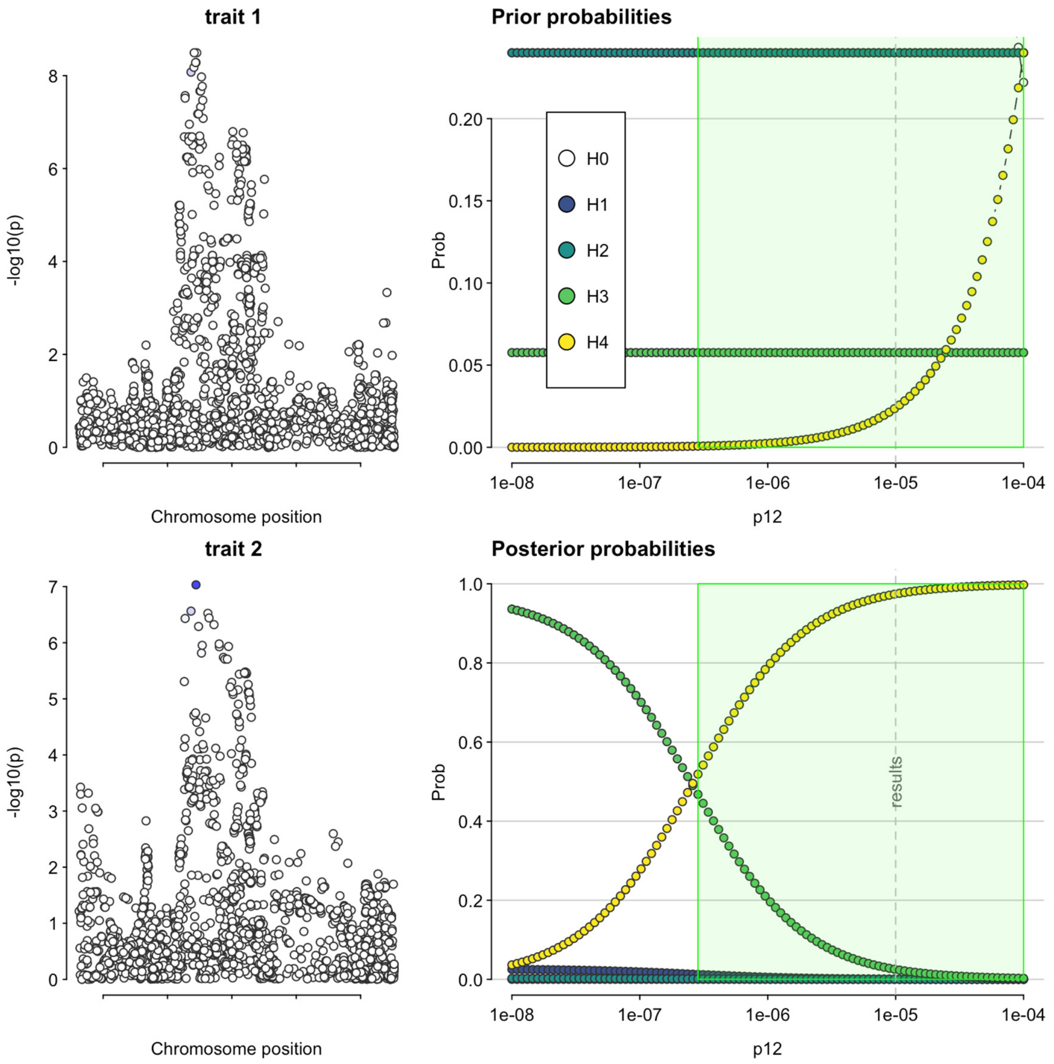

Supplementary Figure 4: *Colocalization for high-density lipoprotein on chromosome 17 between base pairs 7725660 and 8704551. Regions derived from SUPERGNOVA boundaries. The posterior probability of colocalization is 97.3%. Lead SNP rs4791641 posterior probability H_4 of colocalization is 81%. The top left panel is for trait 1 (high-density lipoprotein) and the bottom left panel is for trait 2 (anorexia nervosa), SNP location and association data. SNPs are highlighted based on the magnitude of their posterior probability of H_4, the x-axis indicates genomic location, and the y-axis indicates -log base 10 p value of the SNPs association with the trait. The top right panel indicates the prior probabilities for each hypothesis (H_0-H_4) dependent on the p12 value (prior probability that both traits colocalize). The bottom right panel indicates the posterior probabilities of H_0-H_4 based on p12. The green box indicates the prior and posterior probabilities for p12 where $H_4 > 0.5$. The vertical line labelled 'results' indicates p12 = $1x10^{-5}$, the value that probabilities for H_4 are derived.*

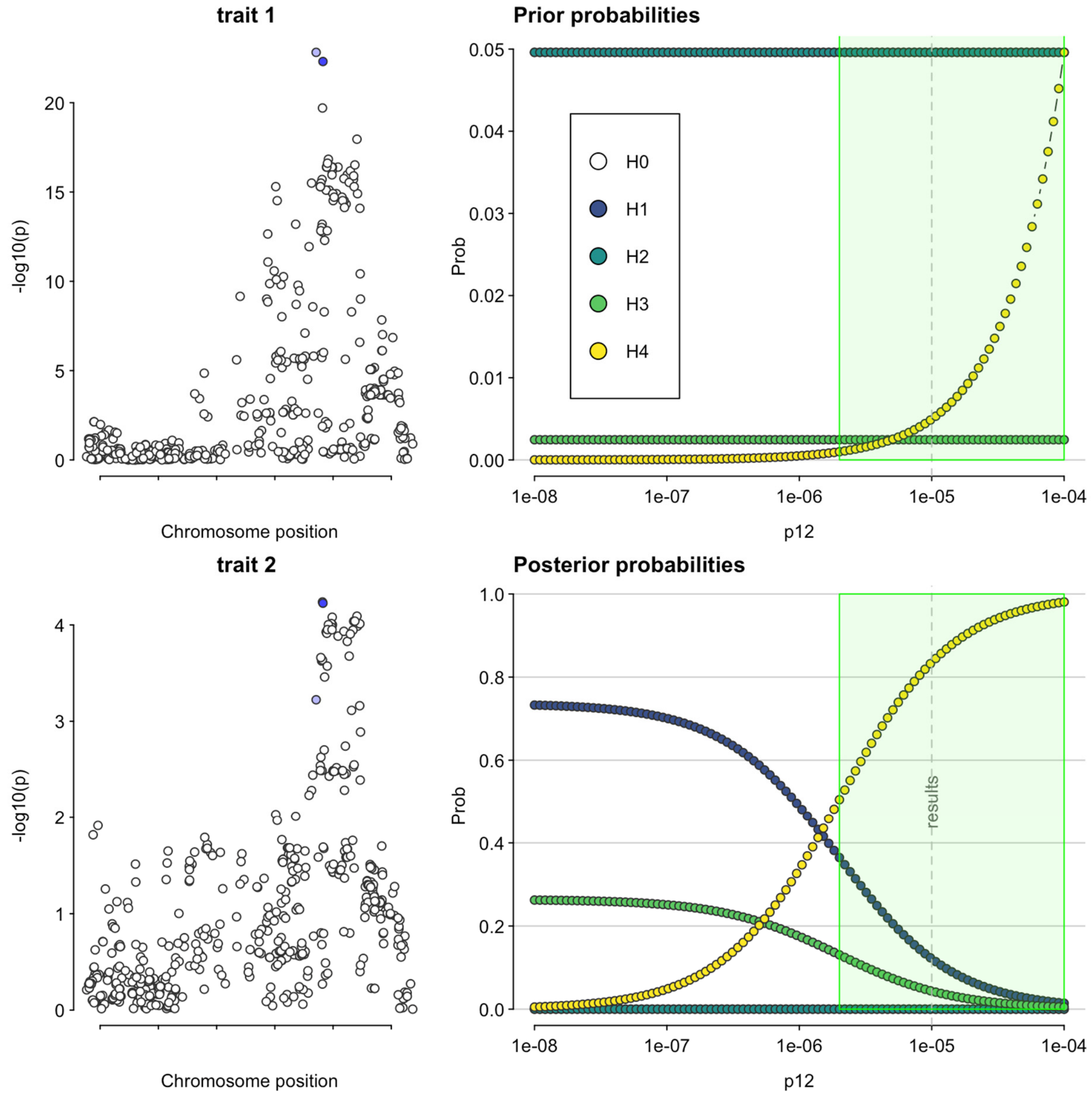

Supplementary Figure 5: *Colocalization for body mass index on chromosome 21 between base pairs 46174981 and 46740019. Regions derived from SUPERGNOVA boundaries. The posterior probability of colocalization is 83.5%. Lead SNP rs397092 posterior probability H_4 of colocalization is 72%. The top left panel is for trait 1 (body mass index), and the bottom left panel is for trait 2 (anorexia nervosa), SNP location and association data. SNPs are highlighted based on the magnitude of their posterior probability of H_4, the x-axis indicates genomic location, and the y-axis indicates -log base 10 p value of the SNPs association with the trait. The top right panel indicates the prior probabilities for each hypothesis (H_0-H_4) dependent on the p12 value (prior probability that both traits colocalize). The bottom right panel indicates the posterior probabilities of H_0-H_4 based on p12. The green box indicates the prior and posterior probabilities for p12 where $H_4 > 0.5$. The vertical line labelled 'results' indicates p12 = $1x10^{-5}$, the value that probabilities for H_4 are derived.*

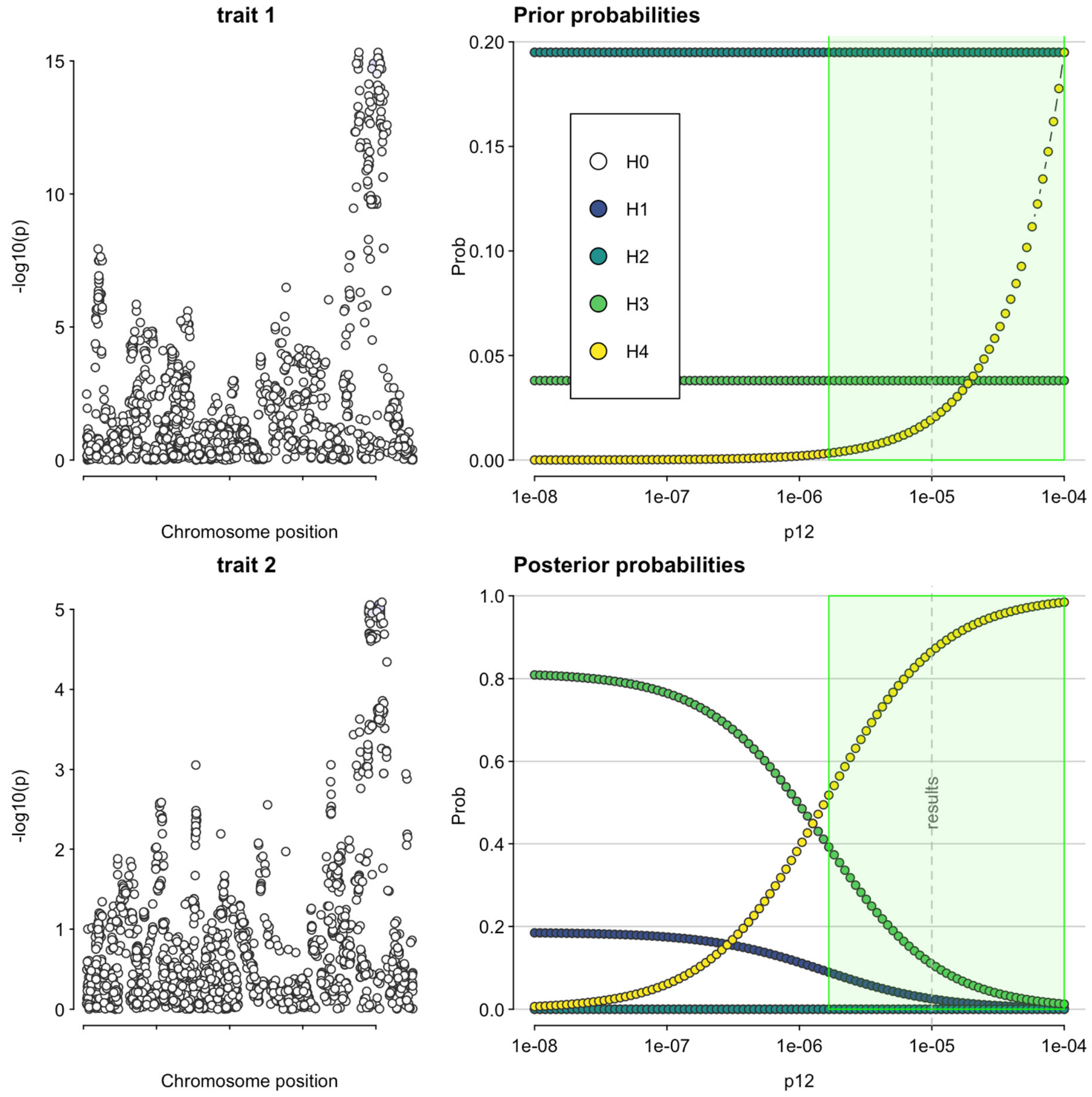

Supplementary Figure 6: *Colocalization for body mass index on chromosome 4 between base pairs: 16521003 and 18752293. Regions derived from SUPERGNOVA boundaries. The posterior probability of colocalization is 86.6%. Lead SNP rs11729200 posterior probability H_4 of colocalization is 24%. The top left panel is for trait 1 (body mass index), and the bottom left panel is for trait 2 (anorexia nervosa), SNP location and association data. SNPs are highlighted based on the magnitude of their posterior probability of H_4, the x-axis indicates genomic location, and the y-axis indicates -log base 10 p value of the SNPs association with the trait. The top right panel indicates the prior probabilities for each hypothesis (H_0-H_4) dependent on the p12 value (prior probability that both traits colocalize). The bottom right panel indicates the posterior probabilities of H_0-H_4 based on p12. The green box indicates the prior and posterior probabilities for p12 where $H_4 > 0.5$. The vertical line labelled 'results' indicates p12 = $1x10^{-5}$, the value that probabilities for H_4 are derived.*

Exploring shared gene and gene-set association signals between anorexia nervosa and metabolic traits

To further explore the genes and biology that may underlie the genetic correlation between AN and metabolic traits, we leveraged the Multi-marker Analysis of GenoMic Annotation (MAGMA) method to assess gene-based and gene-set associations (24). Additionally, we performed a meta-analysis between the gene and gene-set results from the respective MAGMA results for all pairwise comparisons of metabolic traits and AN. Meta-analysing results can provide utility for smaller sample size GWAS, such as leptin and HOMAIR, which may gain additional power to identify risk factors by combining data with a biologically associated trait. When MAGMA is performed for an individual trait, it aggregates the variant level associations for a set of variants localised to a gene (or gene set) to generate a gene (or gene set) based association with the trait of interest. MAGMA was performed to triangulate and support any results from the local genetic correlation and colocalization analysis. Although this process can uncover associations between genetic features and traits through a hypothesis free approach, interpretability of results may be limited due to the presence of confounding. The MAGMA meta-analysis may discover genetic factors that may contribute to shared biological mechanisms, for which the univariate analysis was insufficiently powered. This process functions by aggregating MAGMA gene-based Z scores (probit transformed p values) for each trait using and reweighting these based on the sample size of their relative GWAS such that larger sample size GWAS are upweighted. Thus, increased power within the meta-analysis of gene-association data may uncover sub-threshold genes that pass multiple testing correction in the meta-analysis and may have biological implications for both traits. MAGMA version v1.10 was used with genes and gene-sets defined by the National Center for Biotechnology Information gene definitions and the MSigDB Canonical pathways, respectively (25). Pairwise

meta-analysed genes and gene-sets may indicate functional mechanisms related to the shared biology of AN and the respective metabolic trait.

We applied MAGMA gene and gene-set analysis to six metabolic traits: FI, HOMAIR, leptin, T2D, HDL, BMI, as well as AN *(Supplementary Table 8)* to estimate the association between these factors and trait risk (24). Initially, each trait was analysed on its own (univariate) before being analysed with AN (meta-analysis). Analysis was performed in a genome-wide hypothesis-free manner before exploring genes within prioritised regions. Firstly, we identified 40 significant genes and one pathway associated with AN after multiple-testing correction *(Supplementary Table 5-6)*. This pathway (*Role of ERBB2 (HER2) in Signal Transduction and Oncology* - BioCarta) was unique compared to the pathway previously identified (*Positive Regulation of Embryonic Development* - Gene Ontology Resource) for AN (19), which may be attributed to the updated version of MAGMA utilised. This pathway had no evidence of association with any of the metabolic traits investigated ($P > 0.05$), except for HDL which was nominally associated but did not pass multiple testing correction ($P < 1.76 \times 10^{-4}$). The epidermal growth factor receptor 2 (HER2) pathway is involved in the development of cancers (primarily breast cancer) through controlling cell proliferation and survival (26). However, observational studies report that breast cancer rates (as well as rates of some other cancers) are reduced within individuals with anorexia nervosa (27, 28). It should be noted that the results of MAGMA do not indicate the direction or magnitude of association between genes/pathways and traits, only the significance of the strength of the association. Thus, this may suggest that increased

activation of this *HER2* pathway may reduce risk of anorexia nervosa, while increasing risk of breast cancer.

Across all traits, the largest number of genes and pathways were identified for BMI (1,679 genes and three pathways), followed by for HDL (830 genes and three pathways). No associated pathways were identified for any other traits after multiple-testing correction. In terms of genes, 167 were associated with T2D, while only three were associated with HOMAIR. Interestingly, we identified an overlap between some genes associated with metabolic traits and AN, including BMI (13 genes) and HDL (3 genes). Notably, the 3 genes which overlap AN and HDL also overlap BMI (*TRAIP*, *CDHR4*, *CAMKV*). Briefly, *CAMKV* (CaM Kinase Like Vesicle Associated) is predicted to enable intracellular calcium ion binding and is crucial for dendritic spine maintenance (29). *CDHR4* (Cadherin Related Family Member 4) may be related to cell adhesion by regulating cell morphology, pressure and signalling (30) and *TRAIP* (TRAF Interacting Protein) protects genome stability during replication through DNA repair (31).

MAGMA's meta-analysis was performed for each metabolic trait, meta-analysed against AN gene and gene-set analysis results *(Supplementary Table 7)* (24). The gene-based meta-analysis identified more gene associations in comparison to the univariate analysis which aligned with expectations considering the increased power of this approach. All genes which passed multiple testing correction in the meta-analysis were at least nominally associated with both the metabolic trait and AN (within the univariate analysis) indicating that these genes have some association with both traits. The largest number of novel associations were identified for BMI (64 genes), HDL (42 genes) and T2D (24 genes), which mirrors the proportion of significant associations identified in the univariate trait analysis (Supplementary *Table 8)*. FI and leptin

identified six and two novel associations respectively, while HOMAIR did not identify any. Differences between the number of genes identified may be due to different heritable influences of these traits or shared biological mechanisms with AN. When meta-analysing gene-set association results for both metabolic traits and AN, only two pathways passed multiple testing correction, which did not in the univariate analysis (*Signaling events mediated by Stem cell factor receptor* - Pathway Interaction Database and *Endometrial cancer* - Kyoto Encyclopedia of Genes and Genomes) both for HDL-AN. However, both pathways were only nominally associated with HDL and not AN in the univariate gene-set analysis, which may indicate that these pathways do not directly relate to AN biology. The stem cell receptor (c-kit) has demonstrated roles in cancers such as acute myeloid leukemia and gastrointestinal stromal tumour (32, 33), however has no previously reported association with either AN or HDL biology. Similarly to breast cancer a reduced risk of endometrial cancer has been observed for AN, however this association was not statistically significant (34). AN may exert a protective effect against some cancers, however, there is insufficient evidence to determine if the mechanisms of this association is due to food restriction present in the condition, or underlying biological mechanisms.

To further explore specific genes that may underpin the genetic relationship between AN and metabolic traits, we next integrated MAGMA gene association profiles within regions of local genetic correlation and colocalization data (Supplementary *Figure 7*). Within the meta-analysis gene association results, three genes (*SMARCAD1* - SWI/SNF-Related, Matrix-Associated Actin-Dependent Regulator of Chromatin, Subfamily A, Containing DEAD/H Box 1, *POR* - Cytochrome p450 Oxidoreductase, *MDH2* - Malate Dehydrogenase 2) were associated with BMI-AN, and one gene (*TRPS1* - Transcriptional Repressor GATA Binding 1) was associated with T2D-AN and HDL-AN. Notably, all of these genes reside in loci that exhibit local genetic

correlation between these traits. Interestingly the gene for T2D-AN (*TRPS1*) was prioritised in association with the lead SNP in a region with moderate evidence of colocalization in chromosome 8 (*Table 1*). This gene was nominally associated with both T2D and AN within the univariate MAGMA analysis, however, however only passed multiple testing correction for HDL. The TRPS1 protein regulates gene expression by binding to DNA to influence chromatin accessibility (35) and has previously been associated with dyslipidaemia and coronary artery disease (36, 37). Next, we considered genes associated with AN and metabolic traits from the univariate MAGMA gene analysis, exclusively examining shared genes within regions of local genetics correlation. However, no genes within these regions were associated with both traits after multiple testing correction.

Supplementary Table 5: *Summary table of gene-set association data for metabolic traits and anorexia nervosa.*

TRAIT	*FULL NAME*	*NGENES*	*BETA*	*P*
BMI-AN	REACTOME SIGNALING BY THE B CELL RECEPTOR BCR	113	0.48	$1.77x10^{-05}$
BMI-AN	REACTOME NGF SIGNALLING VIA TRKA FROM THE PLASMA MEMBRANE	130	0.41	$1.46x10^{-04}$
BMI-AN	KEGG THYROID CANCER	28	0.80	$1.69x10^{-04}$
HDL-AN	KEGG LONG TERM DEPRESSION	67	0.65	$2.22x10^{-05}$
HDL-AN	PID AR NONGENOMIC PATHWAY	30	0.86	$3.67x10^{-05}$
HDL-AN	PID KIT PATHWAY	52	0.63	$1.39x10^{-04}$
HDL-AN	KEGG ENDOMETRIAL CANCER	50	0.62	$1.72x10^{-04}$
Leptin-AN	BIOCARTA HER2 PATHWAY	22	0.66	$1.69x10^{-04}$
HDL	KEGG LONG TERM DEPRESSION	67	0.68	$1.08x10^{-05}$
HDL	PID AR NONGENOMIC PATHWAY	30	0.86	$4.42x10^{-05}$
HDL	PID ERBB2 ERBB3 PATHWAY	44	0.67	$9.42x10^{-05}$
BMI	REACTOME SIGNALING BY THE B CELL RECEPTOR BCR	113	0.47	$2.08x10^{-05}$
BMI	REACTOME NGF SIGNALLING VIA TRKA FROM THE PLASMA MEMBRANE	129	0.45	$4.54x10^{-05}$
BMI	KEGG THYROID CANCER	28	0.86	$6.41x10^{-05}$
AN	BIOCARTA HER2 PATHWAY	22	0.70	$1.14x10^{-04}$

Results are for Benjamini-Hochberg significant MAGMA gene-set associations for either univariate metabolic traits or anorexia nervosa (AN) or the meta-analysed results between them. TRAITs indicated as '-AN' have been meta-analysed with AN. Other traits with associations are body mass index (BMI), high-density lipoprotein (HDL) and leptin. Columns indicate the full name of the gene set, ngenes (number of genes in the pathway), BETA (association between the gene set and trait) and P (p value of the BETA estimate). Full results are provided in Supplementary Table 16 – Supplementary Table 22 and Supplementary Table 29 – Supplementary Table 34.

Supplementary Table 6: *Summary table of gene association data for metabolic traits and anorexia nervosa.*

TRAIT	*GENE*	*CHR*	*START*	*NSNPS*	*N*	*ZSTAT*	*P*
Leptin	*FTO*	16	53732875	1843	30931	4.6693	$1.51x10^{-06}$
Leptin	*LOC101928451*	7	128151940	479	30931	4.5048	$3.32x10^{-06}$
Leptin	*ARMT1*	6	151768422	118	30931	3.833	$6.33x10^{-05}$
Leptin	*C19orf48*	19	51299461	49	30931	3.6534	$1.29x10^{-04}$
Leptin	*FIGN*	2	164459947	384	30931	3.625	$1.44x10^{-04}$
HDL	*CTRL*	16	67961973	1	363228	14.933	$1.00x10^{-50}$
HDL	*MT1F*	16	56686855	1	363228	8.2709	$6.65x10^{-17}$
HDL	*ARHGAP1*	11	46697125	7	363228	8.2095	$1.11x10^{-16}$
HDL	*CKAP5*	11	46763584	15	363228	8.1259	$2.22x10^{-16}$
HDL	*DGKZ*	11	46349455	12	363228	8.0988	$2.78x10^{-16}$
HOMAIR	*GCKR*	2	27714470	23	96496	4.736	$1.09x10^{-06}$
HOMAIR	*SPDYE5*	7	74690028	11	96496	4.5781	$2.35x10^{-06}$
HOMAIR	*POM121C*	7	75044560	11	96496	4.5781	$2.35x10^{-06}$
HOMAIR	*PSIP1*	9	15462564	33	96496	4.2946	$8.75x10^{-06}$
HOMAIR	*NFE2L3*	7	26186847	26	96496	3.9716	$3.57x10^{-05}$
T2D	*CDKAL1*	6	20529688	1729	659316	9.9363	$1.45x10^{-23}$
T2D	*WFS1*	4	6266577	119	659316	8.0988	$2.78x10^{-16}$
T2D	*THADA*	2	43456475	692	659316	8.0837	$3.14x10^{-16}$
T2D	*BCAR1*	16	75261428	82	659316	7.6895	$7.38x10^{-15}$
T2D	*KIF11*	10	94347825	163	659316	7.6776	$8.10x10^{-15}$
BMI	*CD19*	16	28938082	1	681275	13.231	$2.90x10^{-40}$
BMI	*NRXN1*	2	50144143	1438	681275	11.254	$1.11x10^{-29}$
BMI	*IL27*	16	28509183	1	681275	10.948	$3.40x10^{-28}$
BMI	*SGCZ*	8	13945873	1878	681275	10.773	$2.32x10^{-27}$
BMI	*RBFOX1*	16	5284469	4254	681275	10.327	$2.67x10^{-25}$
FI	*FTO*	16	53732875	1168	105056	4.7072	$1.26x10^{-06}$
FI	*TCF7L2*	10	114704978	316	105056	4.3625	$6.43x10^{-06}$
FI	*GCKR*	2	27714470	53	105056	4.1734	$1.50x10^{-05}$
FI	*C5orf67*	5	55808192	91	105056	4.0932	$2.13x10^{-05}$
FI	*MYOC*	1	171603057	89	105056	4.0648	$2.40x10^{-05}$
AN	*NCKIPSD*	3	48698919	37	14477	7.6486	$1.02x10^{-14}$
AN	*IP6K2*	3	48723936	53	14477	7.1841	$3.38x10^{-13}$
AN	*C3orf62*	3	49304530	17	14477	7.0834	$7.03x10^{-13}$
AN	*CELSR3*	3	48672396	24	14477	7.0281	$1.05x10^{-12}$
AN	*IMPDH2*	3	49060258	8	14477	6.913	$2.37x10^{-12}$

Results are the top 5 gene association results for each trait based on p value. Traits are FI (fasting insulin), BMI (body mass index), HDL (high-density lipoprotein), HOMAIR (insulin resistance), T2D (type 2 diabetes) and leptin. Column headings are as follows: GENE (name of the gene), CHR (chromosome), START (base pair position at the start of the gene), NSNPS (number of SNPs annotated to the gene), N (sample size), ZSTAT (Z-score derived from p values) and P (gene level p value). Full results are provided in Supplementary Table 9 – Supplementary Table 15.

Supplementary Table 7: *Summary table of gene association data for meta-analysed metabolic traits and anorexia nervosa.*

TRAIT	*GENE*	*CHR*	*START*	*NSNPS*	*N*	*ZSTAT*	*P*
LEPTIN-AN	*SPATS2*	12	49755688	523	45408	4.6997	$1.30x10^{-06}$
LEPTIN-AN	*FTO*	16	53732875	1506	45408	4.6659	$1.54x10^{-06}$
LEPTIN-AN	*QARS*	3	49131865	22	45408	4.5991	$2.12x10^{-06}$
LEPTIN-AN	*LOC101928451*	7	128151940	486	45408	4.5753	$2.38x10^{-06}$
LEPTIN-AN	*QRICH1*	3	49065640	91	45408	4.3654	$6.35x10^{-06}$
HDL-AN	*CTRL*	16	67961973	3	377705	14.622	$1.02x10^{-48}$
HDL-AN	*MST1R*	3	49922935	20	377705	8.5562	$5.83x10^{-18}$
HDL-AN	*MMP9*	20	44632547	27	377705	8.52	$7.98x10^{-18}$
HDL-AN	*AGBL2*	11	47679643	55	377705	8.453	$1.42x10^{-17}$
HDL-AN	*RAPSN*	11	47457808	25	377705	8.3195	$4.42x10^{-17}$
T2D-AN	*CDKAL1*	6	20529688	1930	673793	9.7169	$1.28x10^{-22}$
T2D-AN	*WFS1*	4	6266577	142	673793	7.8494	$2.09x10^{-15}$
T2D-AN	*HMGA2*	12	66213240	184	673793	7.8052	$2.97x10-^{15}$
T2D-AN	*THADA*	2	43456475	798	673793	7.8026	$3.03x10^{-15}$
T2D-AN	*KIF11*	10	94347825	211	673793	7.6876	$7.50x10^{-15}$
HOMAIR-AN	*CAMKV*	3	49893914	22	110973	4.74	$1.07x10^{-06}$
HOMAIR-AN	*TRAIP*	3	49864528	35	110973	4.7294	$1.13x10^{-06}$
HOMAIR-AN	*RNF123*	3	49721950	35	110973	4.7091	$1.24x10^{-06}$
HOMAIR-AN	*GCKR*	2	27714470	37	110973	4.6234	$1.89x10^{-06}$
HOMAIR-AN	*MYOC*	1	171603057	69	110973	4.5408	$2.80x10^{-06}$
BMI-AN	*CD19*	16	28938082	4	695752	13.115	$1.36x10^{-39}$
BMI-AN	*NRXN1*	2	50144143	2450	695752	11.271	$9.12x10^{-30}$
BMI-AN	*IL27*	16	28509183	13	695752	10.988	$2.18x10^{-28}$
BMI-AN	*SGCZ*	8	13945873	3965	695752	10.907	$5.32x10^{-28}$
BMI-AN	*RBFOX1*	16	5284469	8372	695752	10.653	$8.46x10^{-27}$
FI-AN	*TRAIP*	3	49864528	45	119533	5.3241	$5.07x10^{-08}$
FI-AN	*CAMKV*	3	49893914	31	119533	4.9995	$2.87x10^{-07}$
FI-AN	*MST1R*	3	49922935	33	119533	4.914	$4.46x10^{-07}$

FI-AN	*FTO*	16	53732875	1169	119533	4.9135	4.47×10^{-07}
FI-AN	*UBA7*	3	49841138	12	119533	4.8773	5.38×10^{-07}

Results are the top 5 gene association results for each trait pair based on p value. Traits are FI (fasting insulin), BMI (body mass index), HDL (high-density lipoprotein), HOMAIR (insulin resistance), T2D (type 2 diabetes) and leptin. Column headings are as follows: GENE (name of the gene), CHR (chromosome), START (base pair position at the start of the gene), NSNPS (number of SNPs annotated to the gene), N (sample size), ZSTAT (Z-score derived from p values) and P (gene level p value). Full results are provided in Supplementary Table 23 – Supplementary Table 28.

Supplementary Table 8: *Summary of the quantity of associations identified from gene association analysis.*

Trait	*Univariate MAGMA*	*AN MAGMA overlap*	*MAGMA meta-analysis*	*MAGMA meta-analysis unique*
**FI**	1	0	10	6
**T2D**	167	0	184	24
**BMI**	1679	13	1699	64
**Leptin**	1	0	4	2
**HOMAIR**	3	0	4	0
**HDL**	830	3	853	42

Gene association analysis was performed with MAGMA for each of the traits indicated in the first column; high-density lipoprotein (HDL), insulin resistance (HOMAIR), leptin, type 2 diabetes (T2D), fasting insulin (FI) and body mass index (BMI). The number of genes is determined by Bonferroni significance at a 5% alpha threshold. Univariate MAGMA column indicates the number of genes significantly associated with the trait from the univariate analysis. The anorexia nervosa (AN) MAGMA overlap column indicates how many of these genes overlap the 40 genes associated with AN in the univariate analysis. MAGMA meta-analysis indicates the number of genes significantly association with the meta-analysed results between the trait of interest and AN. The unique meta-analysed genes are those that were not significant in the univariate analysis for either the metabolic trait or for AN.

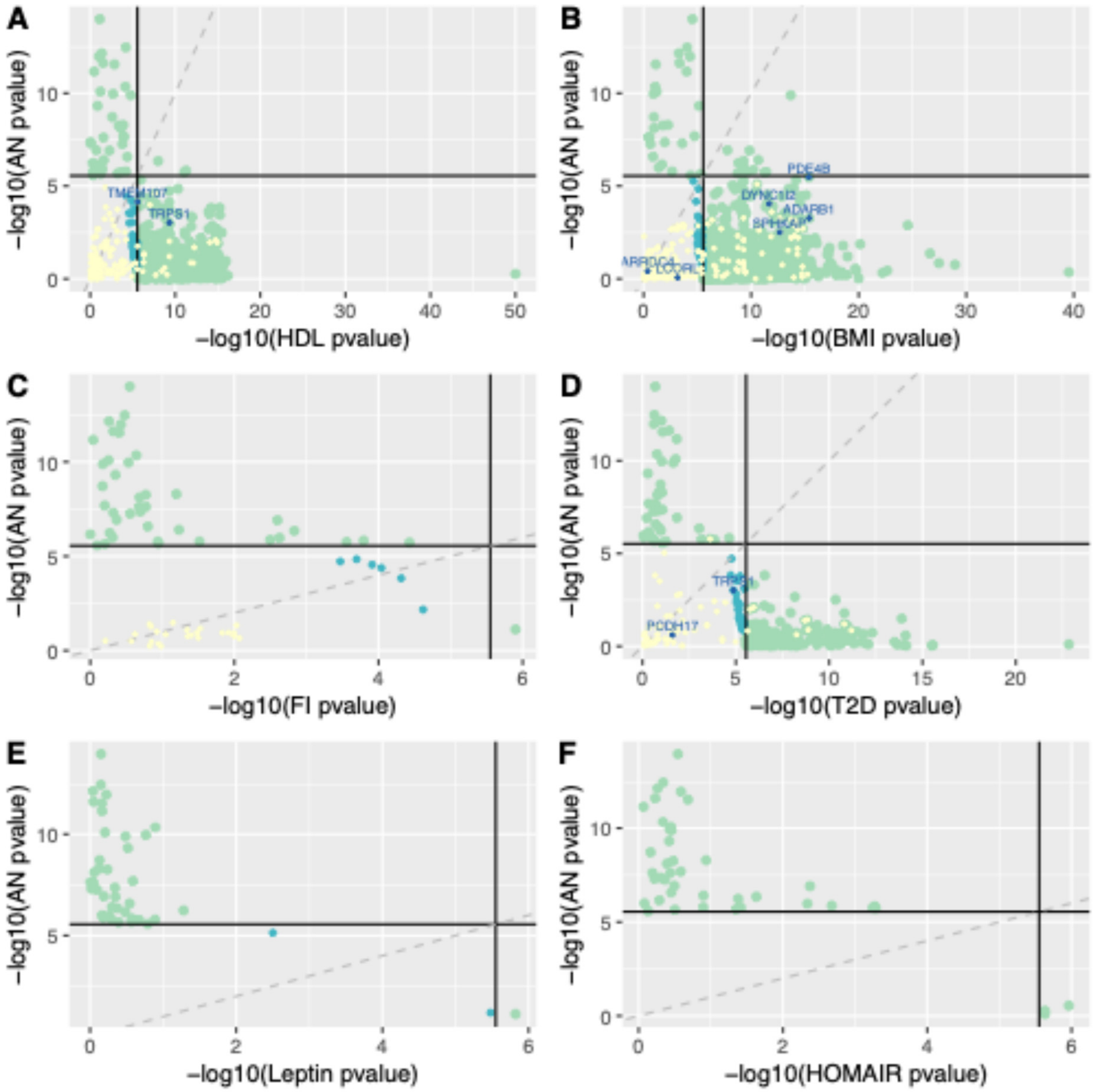

Supplementary Figure 7: *Scatterplots showing the relationship between genes associated with anorexia nervosa (AN) and metabolic traits. Six metabolic traits are indicated **A:** high-density lipoprotein (HDL), **B:** body mass index (BMI), **C:** fasting insulin (FI), **D:** type 2 diabetes (T2D), **E:** leptin and **F:** insulin resistance (HOMAIR). Both axes are scaled to represent a negative log base 10 transformed p value (-log10(p)) for either the metabolic trait on the x-axis or AN on the y-axis. Vertical and horizontal black lines correspond to Bonferroni corrected significance thresholds depending on the number of genes available for each trait. These values ranged between 2.78×10^{-6} and 3.01×10^{-6} for FI and T2D respectively. The dotted diagonal line corresponds to y = x. Gene association estimates were determined with MAGMA gene analysis and meta-analysis. Green points indicate genes which were significantly associated with either trait in the univariate analysis, blue points are those significantly associated in the meta-analysis and yellow points indicate genes which were located within one of the regions for that trait which displayed evidence of significant local genetic correlation (Figure 3). Labelled genes are those which were prioritised from regions with evidence of moderate colocalization (H_4 > 0.4) from COLOC (Table 1).*

References

1. R Core Team (2018). R: A language and environment for statistical computing. R Foundation for Statistical Computing. Vienna, Austria: https://www.R-project.org/.
2. Hemani G, Zheng J, Elsworth B, Wade KH, Haberland V, Baird D, et al. The MR-Base platform supports systematic causal inference across the human phenome. Elife. 2018;7.
3. Burgess S, Butterworth A, Thompson SG. Mendelian randomization analysis with multiple genetic variants using summarized data. Genet Epidemiol. 2013;37(7):658-65.
4. Verbanck M, Chen CY, Neale B, Do R. Detection of widespread horizontal pleiotropy in causal relationships inferred from Mendelian randomization between complex traits and diseases. Nat Genet. 2018;50(5):693-8.
5. Burgess S, Thompson SG. Avoiding bias from weak instruments in Mendelian randomization studies. Int J Epidemiol. 2011;40(3):755-64.
6. Bowden J, Davey Smith G, Burgess S. Mendelian randomization with invalid instruments: effect estimation and bias detection through Egger regression. Int J Epidemiol. 2015;44(2):512-25.
7. Bowden J, Davey Smith G, Haycock PC, Burgess S. Consistent Estimation in Mendelian Randomization with Some Invalid Instruments Using a Weighted Median Estimator. Genetic epidemiology. 2016;40(4):304-14.
8. Hartwig FP, Davey Smith G, Bowden J. Robust inference in summary data Mendelian randomization via the zero modal pleiotropy assumption. Int J Epidemiol. 2017;46(6):1985-98.
9. Bowden J, Del Greco MF, Minelli C, Davey Smith G, Sheehan N, Thompson J. A framework for the investigation of pleiotropy in two-sample summary data Mendelian randomization. Stat Med. 2017;36(11):1783-802.
10. Bowden J, Del Greco M F, Minelli C, Davey Smith G, Sheehan NA, Thompson JR. Assessing the suitability of summary data for two-sample Mendelian randomization analyses using MR-Egger regression: the role of the I2 statistic. International journal of epidemiology. 2016;45(6):1961-74.
11. Bowden J, Del Greco M F, Minelli C, Zhao Q, Lawlor DA, Sheehan NA, et al. Improving the accuracy of two-sample summary-data Mendelian randomization: moving beyond the NOME assumption. International Journal of Epidemiology. 2018;48(3):728-42.
12. Bowden J, Holmes MV. Meta-analysis and Mendelian randomization: A review. Research Synthesis Methods. 2019;10(4):486-96.
13. Slob EAW, Burgess S. A comparison of robust Mendelian randomization methods using summary data. Genetic Epidemiology. 2020;44(4):313-29.
14. Burgess S, Zuber V, Gkatzionis A, Foley CN. Modal-based estimation via heterogeneity-penalized weighting: model averaging for consistent and efficient estimation in Mendelian randomization when a plurality of candidate instruments are valid. Int J Epidemiol. 2018;47(4):1242-54.
15. Burgess S, Foley CN, Allara E, Staley JR, Howson JMM. A robust and efficient method for Mendelian randomization with hundreds of genetic variants. Nature Communications. 2020;11(1):376.
16. Cochran WG. The comparison of percentages in matched samples. Biometrika. 1950;37(3-4):256-66.
17. Bowden J, Hemani G, Davey Smith G. Invited Commentary: Detecting Individual and Global Horizontal Pleiotropy in Mendelian Randomization-A Job for the Humble Heterogeneity Statistic? Am J Epidemiol. 2018;187(12):2681-5.

18. Sanderson E. Multivariable Mendelian Randomization and Mediation. Cold Spring Harb Perspect Med. 2021;11(2).
19. Watson HJ, Yilmaz Z, Thornton LM, Hübel C, Coleman JRI, Gaspar HA, et al. Genome-wide association study identifies eight risk loci and implicates metabo-psychiatric origins for anorexia nervosa. Nature Genetics. 2019;51(8):1207-14.
20. Chen W, Feng J, Jiang S, Guo J, Zhang X, Zhang X, et al. Mendelian randomization analyses identify bidirectional causal relationships of obesity with psychiatric disorders. Journal of Affective Disorders. 2023;339:807-14.
21. Adams DM, Reay WR, Geaghan MP, Cairns MJ. Investigation of glycaemic traits in psychiatric disorders using Mendelian randomisation revealed a causal relationship with anorexia nervosa. Neuropsychopharmacology. 2021;46(6):1093-102.
22. Ding H, Xie M, Wang J, Ouyang M, Huang Y, Yuan F, et al. Shared genetics of psychiatric disorders and type 2 diabetes:a large-scale genome-wide cross-trait analysis. Journal of Psychiatric Research. 2023;159:185-95.
23. Peters T, Antel J, Naaresh R, Laabs BH, Föcker M, Albers N, et al. Suggestive Evidence for Causal Effect of Leptin Levels on Risk for Anorexia Nervosa: Results of a Mendelian Randomization Study. Front Genet. 2021;12:733606.
24. de Leeuw CA, Mooij JM, Heskes T, Posthuma D. MAGMA: generalized gene-set analysis of GWAS data. PLoS Comput Biol. 2015;11(4):e1004219.
25. Subramanian A, Tamayo P, Mootha VK, Mukherjee S, Ebert BL, Gillette MA, et al. Gene set enrichment analysis: a knowledge-based approach for interpreting genome-wide expression profiles. Proc Natl Acad Sci U S A. 2005;102(43):15545-50.
26. Cheng X. A Comprehensive Review of HER2 in Cancer Biology and Therapeutics. Genes (Basel). 2024;15(7).
27. Seminog O, Thakrar DB, James AC, Goldacre MJ. Low risk of some common cancers in women with anorexia nervosa: Evidence from a national record-linkage study. Acta Psychiatrica Scandinavica. 2023;148(1):71-80.
28. Cooper GE, Papini NM, Holde K, Bulik CM, Yilmaz Z, Petersen LV. Eating Disorders and Later Incidence of Cancer: A Nationwide Longitudinal Study in Denmark. Biological Psychiatry Global Open Science. 2025;5(4):100483.
29. Liang Z, Zhan Y, Shen Y, Wong CCL, Yates JR, Plattner F, et al. The pseudokinase CaMKv is required for the activity-dependent maintenance of dendritic spines. Nature Communications. 2016;7(1):13282.
30. Maître JL, Heisenberg CP. Three functions of cadherins in cell adhesion. Curr Biol. 2013;23(14):R626-33.
31. Scaramuzza S, Jones RM, Sadurni MM, Reynolds-Winczura A, Poovathumkadavil D, Farrell A, et al. TRAIP resolves DNA replication-transcription conflicts during the S-phase of unperturbed cells. Nature Communications. 2023;14(1):5071.
32. Sheikh E, Tran T, Vranic S, Levy A, Bonfil RD. Role and significance of c-KIT receptor tyrosine kinase in cancer: A review. Bosn J Basic Med Sci. 2022;22(5):683-98.
33. Lennartsson J, Rönnstrand L. Stem Cell Factor Receptor/c-Kit: From Basic Science to Clinical Implications. Physiological Reviews. 2012;92(4):1619-49.
34. Mellemkjaer L, Papadopoulos FC, Pukkala E, Ekbom A, Gissler M, Christensen J, et al. Cancer Incidence among Patients with Anorexia Nervosa from Sweden, Denmark and Finland. PLOS ONE. 2015;10(5):e0128018.
35. Scott TG, Sathyan KM, Gioeli D, Guertin MJ. TRPS1 modulates chromatin accessibility to regulate estrogen receptor (ER) binding and ER target gene expression in luminal breast cancer cells. bioRxiv. 2023.

36. Adewuyi EO, Porter T, O'Brien EK, Olaniru O, Verdile G, Laws SM. Genome-wide cross-disease analyses highlight causality and shared biological pathways of type 2 diabetes with gastrointestinal disorders. Communications Biology. 2024;7(1):643.
37. Yang L, Gong X, Wang J, Fan Q, Yuan J, Yang X, et al. Functional mechanisms of TRPS1 in disease progression and its potential role in personalized medicine. Pathology - Research and Practice. 2022;237:154022.
